# Influence of disc height and strain-dependent solute diffusivity on metabolic transport in patient-personalized intervertebral disc models

**DOI:** 10.3389/fbioe.2025.1651786

**Published:** 2025-09-05

**Authors:** Zerihun G. Workineh, Estefano Muñoz-Moya, Carlos Ruiz Wills, Dimitrios Lialios, Jérôme Noailly

**Affiliations:** ^1^ BCN MedTech, Department of Engineering, Universitat Pompeu Fabra, Barcelona, Spain; ^2^ Department of Computer Applications in Science and Engineering (CASE), Barcelona Supercomputing Center, Barcelona, Spain

**Keywords:** intervertebral disc, disc morphology, nutrient diffusion, disc material property, cell viability, patient-specific, patient-personalized, finite element

## Abstract

**Introduction:**

Intervertebral disc (IVD) degeneration is a primary contributor to low back pain, with nutritional stress due to the IVD’s avascularity recognized as a key factor. Solute transport within the disc relies predominantly on diffusion, which is governed by tissue morphology and mechanical deformation. However, the interplay between disc geometry, poro-mechanical strain, diffusion, and degeneration remains incompletely characterized. Previous specimen-specific models have captured inter-subject variability in metabolite transport, but the isolated effects of disc height and degeneration-dependent material composition have not been systematically assessed. Moreover, although strain-dependent diffusion coefficients are commonly modeled as porosity functions, the role of intra-element diffusivity gradients (∇D) , arising under large deformation, has been largely overlooked.

**Methods:**

The present study focuses on poro-mechanical finite element (FE) models of three patient-personalized L4-L5 lumbar IVD geometries, representing varying heights categorized as *thin*, *medium*, and *tall* IVDs. Three days of physiological mechanical load cycles, comprising 8 hours of rest and 16 hours of activity, were simulated, under both ’healthy’ (Pfirrmann grade 1) and degenerated (Pfirrmann grade 3) tissue conditions.

**Results:**

Simulation outcomes demonstrated that a one-third reduction in disc height (relative to medium height) led to >30% increases in oxygen and glucose concentrations and ≥20% decreases in lactate levels, particularly in the nucleus and anterior regions. Conversely, a one-third height increase resulted in >30% reductions in oxygen and glucose and a corresponding rise in lactate levels. These deviations were more pronounced in degenerated tissues, highlighting the synergistic role of morphology and matrix integrity in determining metabolic homeostasis. Importantly, the inclusion of ∇D in the diffusion-reaction model produced negligible changes in solute concentration profiles.

**Discussion:**

These findings underscore the predominant influence of disc geometry and matrix composition on IVD metabolic homeostasis, suggesting limited relevance of the (∇D) term in practical simulations. Simplified diffusion models, without (∇D) , may be sufficient for future IVD mechano-transport FE modeling.

## 1 Introduction

The intervertebral disc (IVD) is the largest avascular structure in the human body (Ross Bournemouth, 1931; Humzah and Soames, 1988; Shapiro and Risbud, 2016), contributing significantly to spinal flexibility, load resistance, and distribution along the vertebral column (Humzah and Soames, 1988; Coventry et al., 1945; Newell et al., 2017). It comprises three primary local regions: the nucleus pulposus (NP), annulus fibrosus (AF), and cartilaginous endplates (CEP) (Urban et al., 2000; Ghosh, 2019; Crump et al., 2023). Each region exhibits unique structural, biochemical, and mechanical properties, essential for the disc’s function.

The NP is a gelatinous core predominantly composed of water (approximately 80% by volume), proteoglycans (PGs), and collagen type II (Iatridis et al., 1996; Nedresky et al., 2023; Antoniou et al., 1996; Roughley, 2004). PGs attract and retain water through their negatively charged glycosaminoglycan side chains, which allows the NP to sustain high swelling pressures (Urban and McMullin, 1988). This hydration facilitates the generation of hydrostatic pressure under axial loading, enabling the disc to resist compressive loads and maintain disc height (Urban et al., 2000; McMorran and Gregory, 2021; Molladavoodi et al., 2020). Between the NP and AF lies the transition zone (TZ), a region with mixed characteristics that blends the high PG content of the NP with the increasing collagen content of the AF (Sher et al., 2017). This zone provides a biomechanical and biochemical gradient that contributes to load transfer and helps distribute stress across the NP–AF boundary. The AF surrounds the NP and consists of approximately 15–25 concentric lamellae composed primarily of collagen type I fibers, embedded in a fibrocartilaginous matrix. These fibers are organized in an angle-ply configuration, with alternating fiber orientations in adjacent lamellae, allowing the AF to withstand tensile, shear, and torsional stresses. This structural organization enables the AF to provide mechanical stability, transmit loads, and constrain NP expansion during spinal motion (Ghosh, 2019; McMorran and Gregory, 2021; Molladavoodi et al., 2020; Aladin et al., 2010). The CEP is a thin layer of hyaline cartilage situated at the interface between the IVD and the adjacent vertebral bodies. It serves multiple functions: it anchors the disc to the vertebrae, protects the NP and inner AF from direct mechanical loading, and acts as the primary gateway for nutrient and waste exchange via diffusion from the vertebral capillary beds. Due to the avascular nature of the IVD, the CEP plays a pivotal role in regulating solute transport into the disc (Crump et al., 2023; Wong et al., 2019; Malandrino et al., 2014a; Wu et al., 2021; Buchweitz et al., 2024).

Given the IVD’s avascularity, nutrient transport occurs primarily through diffusion (Katz et al., 1986; Ranganathan et al., 2009; Urban et al., 1977), with the CEP acting as the main conduit for solutes such as oxygen and glucose. These nutrients are essential to sustain disc cell metabolism, and their deficiency has been linked to disc degeneration (Holm et al., 1981; Ishihara and Urban, 1999; Zhu et al., 2012; Bartels et al., 1998; Huang et al., 2014; Jünger et al., 2009; Bibby and Urban, 2004). Inadequate removal of metabolic byproducts, such as lactate, contributes to acidosis and promotes catabolic signaling pathways, thereby exacerbating degeneration (Horner, 2001; Urban, 2002; Bibby and Urban, 2004; Sivan et al., 2014).

While experimental studies have significantly advanced our understanding of IVD physiology and biomechanics, computational modeling has become an indispensable complementary tool. Experimental approaches are often constrained in terms of spatial resolution, repeatability, and the ability to investigate long-term effects or systematically vary patient-personalized (PP) geometries and loading regimes. Computational simulations allow for the controlled exploration of complex biomechanical and biochemical interactions that are difficult to isolate in laboratory settings (Clouthier et al., 2015; Alini et al., 2008; Howard and Masuda, 2006). Finite element (FE) models, in particular, allow for the integration of poromechanical behavior, anisotropic structural features, metabolic reactions, and physiologically relevant mechanical loads (Malandrino et al., 2015a; Gu et al., 2014; Wills et al., 2016; Muñoz-Moya et al., 2024; Lialios et al., 2025). Prior FE studies have examined key parameters influencing nutrient transport, including diffusion path length (Malandrino et al., 2015b), mechanical deformation (Malandrino et al., 2011), solute boundary conditions (Malandrino et al., 2014b), ultrastructural organization (Jackson et al., 2012), osmotic pressurization (Malandrino et al., 2011), and compositional degeneration (Wills et al., 2016; Ruiz Wills et al., 2018).

Organ morphology is now recognized as a critical determinant of nutrient transport efficiency in the IVD. Numerous experimental and computational studies have demonstrated that geometry, particularly disc height and shape, influences diffusion paths and deformation-induced alterations in porosity (Horner, 2001; Urban et al., 1977; Zhu et al., 2012; Jackson et al., 2011; Muñoz-Moya et al., 2024). Tall discs tend to experience nutrient deficiencies due to increased diffusion distances and elevated compressive strain (Malandrino et al., 2015b; Zhu et al., 2012; Muñoz-Moya et al., 2024), contributing to spatial heterogeneity in solute distribution and metabolic stress.

Despite this understanding, a comprehensive investigation of the combined effect of varying degeneration-dependent parameters in composition-dependent constitutive models, along with varying PP IVD geometries, remains pending. Previous computational modeling has either simplified material models for PP geometries or applied advanced constitutive models to generic disc geometries (Malandrino et al., 2014b; Malandrino et al., 2015b; Wills et al., 2016). Furthermore, earlier studies have often lacked systematic, region-specific quantification of solute concentrations across local zones, varying tissue conditions, and physiologically relevant mechanical loading cycles. In addition, the potential influence of strain-induced diffusivity gradients, represented by $\nabla D_{i}$ in the reaction-diffusion formulation, appears to have been overlooked in previous mechano-transport models, despite its possible relevance to solute transport and cell viability.

As we hypothesize that IVD morphology contributes to the risk of initiating or accelerating IVD degeneration, we investigate the interplays among disc morphology, local cell nutrition, nutrition-related cell viability, and degeneration-associated tissue properties. We further develop a new formulation, compatible with state-of-the-art FE solvers that lack multiphysics coupling capabilities, to explore possible biases depending on whether solute transport and mechanical couplings ignore the emergence of strain-induced diffusivity gradients $\left(\nabla D_{i}\right)$ under finite strain.

## 2 Materials and methods

Poro-mechanical Finite Element (FE) models of three different patient-personalized (PP) L4-L5 lumbar IVD models with middle heights of 9 mm (thin), 12 mm (medium), and 16 mm (tall) were considered (Table 1). These three models were selected because they met the degeneration grade threshold ( ≤ Grade III) and offered the most evenly distributed mid-height representation, ensuring a balanced sampling across disc morphologies. These model morphologies are available in the SpineView Intervertebral Disc Database^1^, and were built through a morphing process developed by Muñoz-Moya et al. (2024), using a calibrated and validated FE mesh template (Ruiz et al., 2013; Ruiz Wills et al., 2018) that represented a generic IVD, originally developed by Noailly et al. (2007), Noailly et al. (2011). In brief, the FE meshes were morphed to PP surfaces of the annulus fibrosus and the nucleus pulposus, recreated from segmented patient MRI (Castro-Mateos et al., 2014) using a Bayesian Coherence Point Drift approach (Hirose, 2021) integrated into a custom PP modeling pipeline. Details of this pipeline and the validation thereof can be found in Muñoz-Moya et al. (2024).

**TABLE 1:** Patient-Personalized L4-L5 IVD models, obtained through a morphing process (Muñoz-Moya et al., 2023; Muñoz-Moya et al., 2024), with different morphological factors. Posterior height: PH; Middle height: MH; Anterior height: AH.

Id		PH [mm]	MH [mm]	AH [mm]	SpineView link
GENERIC	-	10.51	14.33	13.69	https://ivd.spineview.upf.edu/?filenamePrefix=GENERIC_L4L5
MY0010	-*thin* (TN)	9.36	8.88	10.89	https://ivd.spineview.upf.edu/?filenamePrefix=MY0010_L4L5
MY0113	-*medium* (MD)	9.12	12.14	14.17	https://ivd.spineview.upf.edu/?filenamePrefix=MY0113_L4L5
MY0097	-*tall* (TL)	7.04	16.17	13.64	https://ivd.spineview.upf.edu/?filenamePrefix=MY0097_L4L5\

**FIGURE 1:**
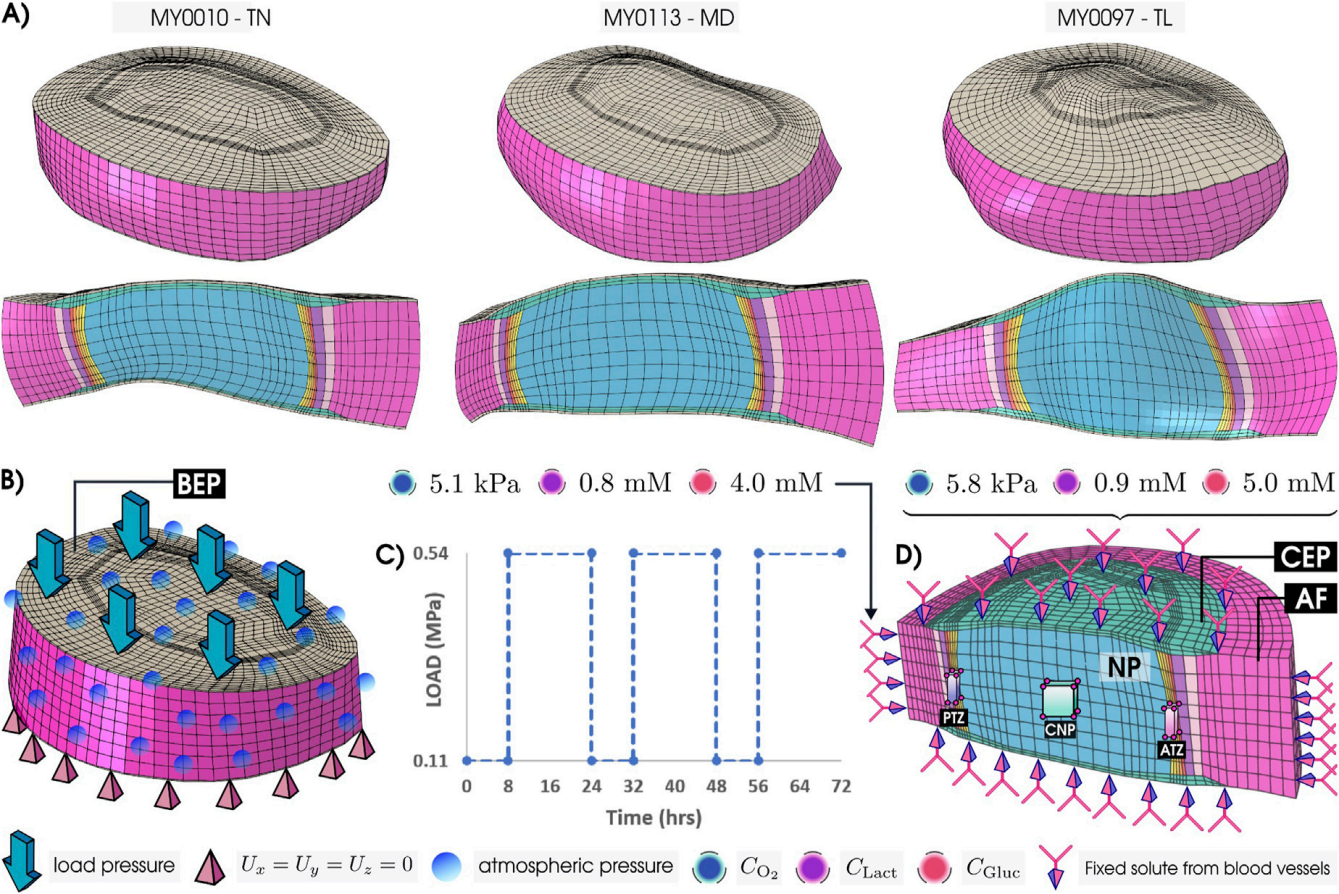
**(A)** Patient-Personalized L4-L5 lumbar IVD models—*Thin* (TN), *Medium* (MD), and *Tall* (TL)—and their sagittal views. **(B)** IVD model under compressive mechanical load on the bony endplate (BEP). **(C)** Mechanical loading variation with time for three consecutive days. **(D)** Coupled nutrient transport and cell viability model with the fixed solute concentrations—oxygen $\left(C_{{\text{O}}_{2}}\right)$ , lactate $\left(C_{\text{Lact}}\right)$ , and glucose $\left(C_{\text{Gluc}}\right)$ —on the outer surfaces of the annulus fibrosis (AF) and the top and bottom cartilage endpalte (CEP). Simulated results reported were averaged over 27 nodes taken from 8 second-order hexahedral elements following Muñoz-Moya et al. (2024). The regions of interest are the posterior transition zone (PTZ), the center of the nucleus pulposus (CNP), and the anterior transition zone (ATZ).

As the IVD model geometries (Figure 1A) were chosen based on mid-height (MH), care was taken to avoid geometries of discs with Pfirrmann degeneration grades higher than grade III. Mechanical models for each morphology (Figures 1B,C) were coupled with a reactive oxygen, glucose, and lactate transport model, and with a corresponding cell viability model (Figure 1D). All simulations were performed using ABAQUS 2023 (Simulia, Providence, RI, United States). The initial nutrient fields of the three models were taken from our Generic model (Table 1) after 3-simulated days of nutrient transport, when it was seen that the metabolic field reached the steady state (Malandrino et al., 2014a; Malandrino et al., 2014b). In this way, all the models begin with the same concentrations in a healthy state, allowing for easy comparison across morphology and material properties.

The selection of target locations—posterior transition zone (PTZ), center of the nucleus pulposus (CNP), and anterior transition zone (ATZ)—aimed to capture solute distribution across spatially distinct regions of the IVD. This choice enables comparison of transport behavior along the antero-posterior axis. Among these, the ATZ has been specifically identified as prone to early degeneration, as shown by clinical imaging in Sher et al. (2017), making it particularly relevant for degeneration-focused analysis. To ensure consistency with previous regional assessments, we selected 27 nodes from eight elements per region, following Muñoz-Moya et al. (2024) (Figure 1), supported by a common mesh structure in all geometrical models, i.e., same mesh topology. The results presented in the manuscript reflect average values across these selected nodes. Additionally, to broaden the assessment beyond PTZ, CNP, and ATZ, we extended the analysis to circumferential paths along the outer surfaces of the NP and transition zone. This approach enables the identification of nutrient-depleted regions and the assessment of how far such areas extend both radially and tangentially across the disc.

The geometrical bounds of the transition zone were determined based on both computational and structural considerations. Initially, this region was introduced in our FE model to ensure mesh convergence and mitigate the fluid flow oscillations generated by weak discontinuities at the interface between the NP and the AF elements (Ruiz et al., 2013). The transition zone, as implemented in our model, thus emerged as a necessary computational feature to maintain numerical stability. However, this region, which had emerged from a need for numerical stabilization, ultimately resulted in a sound definition of a local tissue region, as several studies using quantitative MRI, synchrotron imaging, and compositional analyses had demonstrated the presence of a structurally distinct transition zone between the NP and AF (Bruehlmann et al., 2002; Marchand and Ahmed, 1990; Disney et al., 2022).

The sequential numerical scheme employed by Malandrino et al. (2011), Malandrino et al. (2014b) has been adopted in this study. This implementation integrates a poro-mechanical model with transport FE models, capturing the complex interplay of metabolic reactions within the intervertebral disc. Each solute transport model leverages the deformation history from the poro-mechanical model to dynamically update concentration gradients over time. At each time step, the transport models are solved sequentially, incorporating a cell viability model that adjusts cell density and, in turn, modulates solute consumption and production. For a more detailed illustration of this framework, please refer to the schematic diagram presented in Supplementary Section S1 (Numerical Implementation) of the Supplementary Information.

### 2.1 Constitutive modeling of the IVD

#### 2.1.1 Mechanical model

The constitutive models of the NP, AF, and CEP tissues combined the poro-mechanical interactions among: a hyperelastic porous matrix; intra- and extra-fibrillar interstitial fluid; a Donnan osmotic pressure; viscoelastic collagen fibers (Wills et al., 2016; Schroeder et al., 2007; Cortes et al., 2014). The total stress tensor (Equation 1), σ , in each tissue, was the additive contribution of the effective stress, $\sigma_{\text{eff}}$ , of the porous solid part and a pore pressure, p (Wills et al., 2016; Schroeder et al., 2007):

$$\sigma =\sigma_{\text{eff}}-pI,$$ (1)

where, I is the identity matrix. p=μ+Δπ represents the combined effect of the water chemical potential, μ , and the osmotic pressure, Δπ . $\sigma_{\text{eff}}$ was described through a neo-Hookean model (Equation 2), modified for large tissue consolidation (Schroeder et al., 2008):

$$\sigma_{\text{eff}}=-\frac{ln\left(J\right)}{2J}G_{m}I\left[-\frac{1}{3}+\frac{J+1-n_{f,0}}{-J+1-n_{f,0}}+\frac{Jln\left(J\right)\left(1-n_{f,0}\right)}{{\left(-J+1-n_{f,0}\right)}^{2}}\right]+\frac{G_{m}}{J}\left(B-J^{2/3}I\right),$$ (2)

where J is the determinant of the deformation gradient tensor F , $G_{m}$ is the shear modulus, $n_{f,0}$ is initial water content (porosity), and B is the left Cauchy–Green strain tensor. The osmotic pressure model (Equation 3) was assumed to have equilibrated ion concentrations, and the osmotic pressure gradient was calculated as follow Schroeder et al. (2007); Wilson et al. (2005):

$$\Delta \pi =RT\left[\phi_{\mathrm{int}}\sqrt{c_{F}^{2}+4{\left(\frac{\gamma_{\mathrm{ext}}^{\pm }}{\gamma_{\mathrm{int}}^{\pm }}\right)}^{2}c_{\mathrm{ext}}^{2}}-2\phi_{\mathrm{ext}}c_{\mathrm{ext}}\right].$$ (3)

Where, R is gas constant, T is the absolute temperature, $\phi_{\mathrm{int}}$ , and $\phi_{\mathrm{ext}}$ are internal and external osmotic coefficients, respectively. $c_{\mathrm{ext}}$ is the external salt concentration, $c_{F}$ (Schroeder et al., 2007) is the fixed charge density per total water volume, and $\gamma_{\mathrm{int}}$ and $\gamma_{\mathrm{ext}}$ are internal and external activity coefficients (Wilson et al., 2005), respectively. The water chemical potential, μ , in our study was simply the pore pressure, locally updated out of the fluid velocity, according to Darcy’s law. The term “water chemical” potential was adopted after the work of Huyghe et al. (2004). It is calculated through Darcy’s relation between the spatial gradient of μ and the interstitial fluid velocity, v , in the porous solid, following Equation 4:

$$vn_{f}=-k\nabla \mu ,$$ (4)

with $n_{f}$ be current porosity (fluid volume fraction in the saturated porous solid) and k is the hydraulic permeability (Equation 5), expressed as a function of extra fibrillar fluid fraction, $n_{\mathrm{exf}}$ (Schroeder et al., 2007);

$$k=k_{0}{\left(1-n_{\text{exf}}\right)}^{-M},$$ (5)

where $k_{0}$ is the initial permeability and M is a positive constant. The current porosity or water content $n_{f}$ is expressed in terms of J as:

$$n_{f}=\frac{n_{f,0}+J-1}{J}$$ (6)

Key Pfirrmann grade-dependent parameter values for ‘healthy’ state, corresponding to Pfirrmann grade I (GR1), and a degenerated state, corresponding to Pfirrmann grade III (GR3), includes initial water content, shear modulus, fixed charge density, collagen content, and others, are adopted from the references Wills et al. (2016); Barthelemy et al. (2016) and are detailed in Table 2 for all tissues considered.

**TABLE 2:** Summary of key parameters employed in the simulation for GR1 and GR3 material properties across all three tissue types (Wills et al., 2016; Ruiz Wills et al., 2018; Malandrino et al., 2011; Barthelemy et al., 2016).

Initial parameter	Tissue					
	NP		AF		CEP	
	GR1	GR3	GR1	GR3	GR1	GR3
Shear modulus ( $G_{m}$ ) (MPa)	1	0.8	1.0	0.8	1.0	0.8
Water content ( $n_{f,0}$ ) (%)	80	76	75	70	66	60
Charge density ( $c_{F}$ ) ( $mEqmL^{-1}$ )	0.3	0.23	0.2	0.2	0.17	0.13
Dry weight ( $\rho_{c}$ ) (%)	15	28.5	65	78	24	35
External salt ( $c_{\mathrm{ext}}$ ) ( $mEqmL^{-1}$ )	0.15	0.15	0.15	0.15	0.15	0.15
Permeability ( $k_{0}$ ) ( $mm^{4}N^{-1}s^{-1}$ )	0.00016	0.00045	0.00016	0.00045	0.00017	0.00044
M	8.5	8.5	1.18	1.18	4.63	4.63

#### 2.1.2 Solute transport model

Reactive solute transport model, which is coupled to tissue deformation and osmosis, has been modelled by a reaction-diffusion equation as:

$$\frac{\partial }{\partial t}\left(\begin{array}{c} C_{{\text{O}}_{2}}\\ C_{\text{Lact}}\\ C_{\text{Gluc}} \end{array}\right)+\nabla \cdot \left(\begin{array}{c} f_{{\text{O}}_{2}}\\ f_{\text{Lact}}\\ f_{\text{Gluc}} \end{array}\right)=\left(\begin{array}{c} S_{{\text{O}}_{2}}\\ S_{\text{Lact}}\\ S_{\text{Gluc}} \end{array}\right)$$ (7)

with, $f_{i}$ is the flux of oxygen, lactate, and glucose $\left(i\equiv {\text{O}}_{2},\text{Lact},\text{Gluc}\right)$ and $\nabla \cdot f_{i}$ is its divergence. Their respective expressions are given in Equations 8, 9.

$$f_{i}=-D_{i}\nabla C_{i}$$ (8)

$$\nabla \cdot f_{i}=\nabla \cdot \left(-D_{i}\nabla C_{i}\right)=-\nabla D_{i}\cdot \nabla C_{i}-D_{i}\nabla^{2}C_{i}$$ (9)

where, $S_{i}$ is rate of solute reactions. $D_{i}$ and $C_{i}$ are strain-dependent diffusivity and concentration of solute i , respectively. Substituting Equation 9 in Equation 7 gives the reaction diffusion equation of:

$$\frac{\partial C_{i}}{\partial t}-\nabla D_{i}\cdot \nabla C_{i}-D_{i}\nabla^{2}C_{i}=S_{i}$$ (10)

Equation 10 highlights that reactive diffusion of a solute depends not only on its diffusivity but also on the gradient of diffusivity. This contrasts with previous implementations in ABAQUS, where heat transfer elements were employed to simulate diffusive transport (Ferguson et al., 2004; Galbusera et al., 2013; Malandrino et al., 2011; Malandrino et al., 2014b; Malandrino et al., 2014a; Malandrino et al., 2015b). As noted earlier, the heat transfer elements typically utilized in solvers like ABAQUS lack built-in capabilities to calculate the spatial derivatives of strain-dependent diffusivity. Consequently, $\nabla D_{i}$ is derived analytically to enable the solution of Equation 10. The effective strain-dependent diffusivity $D_{i}$ (Equation 11) is calculated using the Mackie-Meares equation $D_{\text{MM}}$ (Mackie et al., 1955a; Mackie et al., 1955b; Wills et al., 2016), relating the solute’s volume-averaged isotropic diffusivity to the tissue water content $n_{f}$ ( $\equiv \text{porosity},n_{f}$ in Equation 6) and its water diffusivity $D_{w_{i}}$ :

$$D_{i}=D_{\text{MM}}=D_{w_{i}}{\left(\frac{n_{f}}{2-n_{f}}\right)}^{2}$$ (11)

The derivation of the spatial gradient of the effective diffusivity D in a deforming porous medium is:

$$\nabla D_{i}=D_{w_{i}}\cdot \frac{4n_{f}\left(1-n_{f}\right)}{J{\left(2-n_{f}\right)}^{3}}\cdot \nabla J$$ (12)

The three free water diffusivity values $D_{w_{i}}$ for glucose, lactate, and oxygen at a body temperature (≈37°C) are $9.167\times 10^{-4}{\text{mm}}^{2}s^{-1}$ , $1.39\times 10^{-3}{\text{mm}}^{2}s^{-1}$ and $3.0\times 10^{-3}{\text{mm}}^{2}s^{-1}$ , respectively (Magnier et al., 2009). J=det(F) is the Jacobian of the deformation gradient. ∇J is the spatial gradient of J . Using Jacobi’s formulation, the gradient of J is given by:

$$\begin{array}{cc} \nabla J=\nabla \det F & =\det F\cdot \mathrm{tr}\left(F^{-1}\cdot \nabla F\right)\\ & =J\cdot \mathrm{tr}\left(F^{-1}\cdot \nabla F\right) \end{array}$$ (13)

Substituting Equation 13 into Equation 12, Equation 14 is obtained:

$$\nabla D_{i}=D_{w_{i}}\cdot \frac{4n_{f}\left(1-n_{f}\right)}{{\left(2-n_{f}\right)}^{3}}\cdot \mathrm{tr}\left(F^{-1}\cdot \nabla F\right)$$ (14)

Considering the gradient (Equation 15) in the current configuration, $\nabla D_{i}=\partial D_{i}/\partial x_{k}$ :

$$\left.\begin{array}{c} \frac{\partial D_{i}}{\partial x_{k}}=D_{w_{i}}\;\frac{4n_{f}\;\left(1-n_{f}\right)}{{\left(2-n_{f}\right)}^{3}}{ϒ}_{k};\;\text{with}k=\left\{1,2,3\right\},\;\\ \to \underset{{ϒ}_{k}={\mathrm{tr}}_{k}\;\left(F^{-1}\partial_{x_{k}}F\right)}{\underbrace{ϒ=\;\left[\begin{array}{c} \mathrm{tr}\;\left(F^{-1}\;\partial_{x}F\right)\\ \mathrm{tr}\;\left(F^{-1}\;\partial_{y}F\right)\\ \mathrm{tr}\;\left(F^{-1}\;\partial_{z}F\right) \end{array}\right]}}\;\Rightarrow \;{ϒ}_{k}=\underset{\text{ Trace cyclicity}}{\underbrace{\overset{3}{\underset{a=1}{\sum }}\overset{3}{\underset{b=1}{\sum }}F_{ab}^{-1}\;{\left(\partial_{x_{k}}F\right)}_{ba}}},\;\\ \to \partial_{x_{k}}F=\overset{20}{\underset{A=1}{\sum }}u^{A}⊗\frac{\partial }{\partial x_{k}}\left(\nabla_{X}N^{A}\right)\;\\ \;=\overset{20}{\underset{A=1}{\sum }}u^{A}⊗\frac{\partial^{2}N^{A}}{\partial x_{k}\partial X_{n}};\;\text{with}n=\left\{1,2,3\right\}.\; \end{array}\right\}$$ (15)

The second mixed partial derivative of the shape functions (Equation 16) requires the pull-back of contravariant indices to express it in terms of the current x and reference X configurations:

$$\frac{\partial^{2}N^{A}}{\partial x_{k}\partial X_{n}}=\underset{\begin{array}{c} \text{ Pull}-\text{back of}\\ \text{ contravariant index }k \end{array}}{\underbrace{\overset{3}{\underset{m=1}{\sum }}F_{mk}^{-1}\frac{\partial^{2}N^{A}}{\partial X_{m}\partial X_{n}}}},\text{with}{\;F}_{km}=\frac{\partial x_{k}}{\partial X_{m}}.$$ (16)

The second derivative of the shape functions (Equation 17) with respect to the reference coordinates is computed via the chain rule as:

$$\frac{\partial^{2}N^{A}}{\partial X_{m}\partial X_{n}}=\overset{3}{\underset{p,q=1}{\sum }}\left(\frac{\partial^{2}N^{A}}{\partial \xi_{p}\partial \xi_{q}}\frac{\partial \xi_{p}}{\partial X_{m}}\frac{\partial \xi_{q}}{\partial X_{n}}\right)+\overset{3}{\underset{p=1}{\sum }}\frac{\partial N^{A}}{\partial \xi_{p}}\frac{\partial^{2}\xi_{p}}{\partial X_{m}\partial X_{n}}$$ (17)

The mapping from the isoparametric ξ to the reference frame X is described in Equation 18 by the Jacobian matrix $\underset{\to }{J_{\xi X}}={\left[\underset{\to }{J_{\xi \;X}}\right]}_{mp}=\partial X_{m}/\partial \xi_{p}$ :

$$\frac{\partial {\left[\underset{\to }{J_{\xi \;X}^{-1}}\right]}_{pq}}{\partial X_{t}}=\underset{\text{ Inverse matrix derivative identity.}}{\underbrace{-\overset{3}{\underset{r^{\prime },s^{\prime }=1}{\sum }}{\left[\underset{\to }{J_{\xi \;X}^{-1}}\right]}_{pr^{\prime }}\frac{\partial {\left[\underset{\to }{J_{\xi \;X}}\right]}_{r^{\prime }s^{\prime }}}{\partial X_{t}}{\left[\underset{\to }{J_{\xi \;X}^{-1}}\right]}_{s^{\prime }q}}}$$ (18)

We can compute the second derivatives (Equation 19) entirely in reference coordinates:

$$\left.\begin{array}{c} \begin{array}{cc} \frac{\partial^{2}N^{A}}{\partial X_{m}\partial X_{n}}= & \overset{3}{\underset{p,q=1}{\sum }}\frac{\partial^{2}N^{A}}{\partial \xi_{p}\partial \xi_{q}}{\left[\underset{\to }{J_{\xi \;X}^{-1}}\right]}_{pm}{\left[\underset{\to }{J_{\xi \;X}^{-1}}\right]}_{qn}\\ & +\overset{3}{\underset{p=1}{\sum }}\frac{\partial N^{A}}{\partial \xi_{p}}\underset{\text{Clairaut’s theorem}}{\underbrace{\left(\frac{\partial {\left[\underset{\to }{J_{\xi \;X}^{-1}}\right]}_{pm}}{\partial X_{n}}+\frac{\partial {\left[\underset{\to }{J_{\xi \;X}^{-1}}\right]}_{pn}}{\partial X_{m}}\right)}}\; \end{array} \end{array}\right\}$$ (19)

Here, $\left(x,y,z\right)\equiv \left(x_{1},x_{2},x_{3}\right)$ denote spatial indices k , whereas $\left(X_{1},X_{2},X_{3}\right)$ are reference indices m . Because the deformation gradient is defined with respect to the reference configuration, $F=I+\sum_{A}u^{A}\left(\text{nodal disp.}\right)⊗\nabla_{X}N^{A}$ , its spatial gradient naturally involves the mixed second derivative $\partial^{2}N^{A}/\partial x_{k}\partial X_{n}$ . The shape functions $N^{A}$ are polynomials of class $C^{2}\left(\hat{\Omega }\right)$ on the parent domain $\hat{\Omega }$ , so mixed partial derivatives commute and those second-order terms are well defined. In short, ∇J (Equation 12) quantifies the spatial heterogeneity of the volume change: where it is large, the porosity (and hence the effective diffusivity) varies sharply, whereas where it is zero, the deformation is uniform and no additional diffusive driving force is introduced.

The metabolic reaction term $\left(S_{i}\right)$ , which represents the cell consumption rate of oxygen and glucose and the production rate of lactate, primarily depends on the concentration of oxygen $\left(C_{{\text{O}}_{2}}\right)$ and pH values (Bibby et al., 2005; Wills et al., 2016), as given by:

$$S_{{\text{O}}_{2}}=-\frac{n_{f}\;\rho_{\mathrm{cell}}}{3600\;{\text{Sol}}_{{\text{O}}_{2}}}\left(\frac{7.20\;C_{{\text{O}}_{2}}\left(\text{pH}-4.95\right)}{1.46+C_{{\text{O}}_{2}}+4.03\left(\text{pH}-4.95\right)}\right)$$ (20)

and

$$S_{\text{Lact}}=\frac{\rho_{\mathrm{cell}}}{3600}\exp\left[-2.47+0.93\text{pH}+0.16C_{{\text{O}}_{2}}-0.0058C_{{\text{O}}_{2}}^{2}\right]$$ (21)

Here, $C_{{\text{O}}_{2}}$ is expressed in kPa , while $C_{\text{Lact}}$ is in mM . The unit of $S_{\text{Lact}}$ (Equation 12) is $\text{mM}\cdot {\text{s}}^{-1}$ , and $S_{{\text{O}}_{2}}$ (Equation 11) is $\text{kPa}\cdot {\text{s}}^{-1}$ . The solubility of oxygen in water, $Sol_{{\text{O}}_{2}}$ , is $1.0268\times 10^{-2}\left[\text{mM}\cdot {\text{kPa}}^{-1}\right]$ . Cell consumption rate of glucose can be estimated as half of the lactate production rate: $S_{\text{Gluc}}=-\frac{1}{2}S_{\text{Lact}}\left(\text{mM}\cdot {\text{s}}^{-1}\right)$ (Bibby et al., 2005). pH is also related to $C_{\text{Lact}}$ as $\text{pH}=7.4-0.09C_{\text{Lact}}$ (Bibby et al., 2005). The boundary concentration values of all solutes and initial cell densities (Wills et al., 2016) are listed in Table 2. The initial nutrient fields of the three models were taken from our Generic model (Table 1) after 3-simulated days of nutrient transport, when it was seen that the metabolic field reached the steady state (Malandrino et al., 2014b; Malandrino et al., 2014a). In this way, all the models begin with the same concentrations in a healthy state, allowing for easy comparison across morphology and material properties.

**TABLE 3:** Boundary concentration values (graphically represented in Figure 1) and initial cell densities $\left(\rho_{\text{cell},0}\right)$ of the annulus fibrosis (AF), nucleus pulposus (NP), and the cartilage endplate (CEP).

Values	$C_{{\text{O}}_{2}}\left[\text{kPa}\right]$	$C_{\text{Gluc}}\left[\text{mM}\right]$	$C_{\text{Lact}}\left[\text{mM}\right]$	$\rho_{\text{cell},0}$ , NP	$\rho_{\text{cell},0}$ , AF	$\rho_{\text{cell},0}$ , CEP
Boundary, CEP	5.1	4.0	0.8			
Boundary, AF	5.8	5.0	0.9			
Cell density [ $10^{6}$ cells/ ${\text{mm}}^{3}$ ]				0.0036	0.0055	0.0135

#### 2.1.3 Cell viability

The rates of consumption-sink ( $S_{{\text{O}}_{2}}$ and $S_{\text{Gluc}}$ ) and production-source $\left(S_{\text{Lact}}\right)$ are dependent on cell density $\left(\rho_{\text{cell}}\right)$ , which, in turn, is influenced by glucose concentration (Horner, 2001; Bibby et al., 2005). Horner (2001) demonstrated that intervertebral disc NP cells can survive under extremely low oxygen levels but are highly sensitive to reduced glucose concentrations and acidic environments. As a result, models (Equation 22) of cell viability (cell%) integrate the effects of both glucose concentration and pH levels into their formulations (Zhu et al., 2012; Malandrino et al., 2014b), expressed as follows:

$$\text{cell}\%=\frac{\rho_{\text{cell}}}{\rho_{\text{cell},0}}J={\text{e}}^{\left[\alpha_{\text{decay}}\Delta t\right]}$$ (22)

Where Δt is the time since cell death was initiated. The decay coefficient $\alpha_{\text{decay}}$ ${\text{s}}^{-1}$ accounts for glucose $\left(\alpha_{\text{Gluc}}\right)$ and pH $\left(\alpha_{\text{pH}}\right)$ effects (Zhu et al., 2012; Malandrino et al., 2015a):

$$\left.\begin{array}{c} \begin{array}{cc} \alpha_{\text{decay}}= & \alpha_{\text{Gluc}}+\alpha_{\text{pH}}\\ \alpha_{\text{Gluc}}= & \left\{\begin{array}{cc} \begin{array}{cc} \alpha_{\text{t}} & \left(\frac{\left(C_{\text{Gluc}}-C_{\text{Gluc,T}}\right)}{C_{\text{Gluc}}+K}-\right.\\ & \left.\frac{\left|C_{\text{Gluc}}-C_{\text{Gluc,T}}\right|}{C_{\text{Gluc}}+K}\right) \end{array},\; & \text{if }C_{\text{Gluc}}<C_{\text{Gluc,T}},\\ 0,\; & \text{otherwise}, \end{array}\right.\\ \alpha_{\text{pH}}= & \left\{\begin{array}{cc} -3.43\times 10^{-6},\; & \text{if }\text{pH}<{\text{pH}}_{\text{T}},\\ 0,\; & \text{otherwise}. \end{array}\right. \end{array} \end{array}\right\}$$ (23)

where $C_{\text{Gluc,T}}=0.5$ mM and ${\text{pH}}_{\text{T}}=6.78$ represent glucose and pH survival thresholds, K=0.2 mM with $\alpha_{\text{t}}=1{\text{ day}}^{-1}=1/86400{\text{ s}}^{-1}$ (Bibby et al., 2002; Horner, 2001; Zhu et al., 2012).

### 2.2 Model verification

To verify our cell viability model (Equation 23) coupled with our diffusion-reaction model (Equation 10), a diffusion chamber was simulated following experimental tests on bovine nucleus cell viability (Horner, 2001). A 26 mm width diffusion chamber filled with cells embedded in 1% agarose gel was modeled. Diffusivities of oxygen, glucose, and lactate in water were modified to account for gel porosity of $n_{f,gel}=0.95$ by using Mackie and Meares formulation (Mackie et al., 1955a; Wills et al., 2016). Initial and boundary conditions were applied throughout the chamber for the three metabolites to reproduce the *in vitro* experiment (Horner, 2001). Initial and boundary values of pH 7.4 (initial null lactate concentration), oxygen 21 kPa, and glucose 5 mM were considered. The comparison was conducted for three distinct cell densities (2, 4, and 8 million cells per $cm^{3}$ ), with cell viability evaluated after 3 and 11 simulated days, corresponding to the experimental measurements.

### 2.3 Data analysis

#### 2.3.1 Effect of diffusivity gradient

To evaluate the influence of a spatial gradient in diffusivity (∇D) on solute transport, we calculated the relative change in solute concentrations as:

$$\text{Effect of }\nabla D\left(\%\right)=\frac{C_{\nabla D\neq 0}-C_{\nabla D=0}}{C_{\nabla D=0}},$$ (24)

where $C_{\nabla D=0}$ and $C_{\nabla D\neq 0}$ represent solute concentrations computed without and with the inclusion of the ∇D=0 term, respectively.This metric provides a quantitative assessment of whether incorporating the strain-induced diffusivity gradient into the reaction-diffusion model (Equation 10) produces a significant impact on solute concentrations across the regions of interest, PTZ, CNP, and ATZ, under all combinations of material properties (GR1 and GR3) and mechanical loading conditions.

#### 2.3.2 Spatio-temporal distribution of solutes under varying material properties

The spatial distribution and temporal evolution of solute concentrations were analyzed using a set of quantitative approaches designed to assess the effects of material property variation. Specifically, the analysis considered two distinct sets of material properties representing different disc conditions: a healthy disc modeled as Pfirrmann grade I (GR1), and a degenerated disc modeled as Pfirrmann grade III (GR3). To characterize regional solute dynamics, we first examined spatial and temporal variations over a 72-h simulation period across three locally distinct regions. In each region, solute concentrations were averaged over 27 nodes sampled from eight mesh elements to maintain consistent spatial resolution across all models (Figure 1). To isolate the influence of tissue degeneration, the relative change in solute concentration between GR3 and GR1 conditions was quantified as:

$$\text{Effect of }\text{Material}\;\text{Property}\left(\%\right)=\frac{C_{i,\text{GR}3}-C_{i,\text{GR}1}}{C_{i,\text{GR}1}},$$ (25)

where $C_{i,\text{GR}1}$ and $C_{i,\text{GR}3}$ represent the concentration of solute i under GR1 and GR3 material properties, respectively.

**FIGURE 2:**
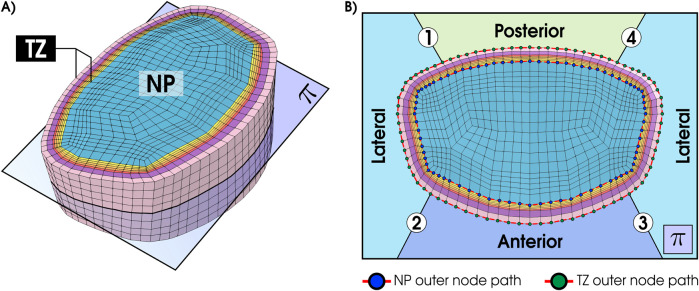
**(A)** Mid-transverse plane π cutting the NP and TZ. **(B)** View in plane π showing the external nodal path and the four segments: Lateral-1 (1 → 2), Anterior (2 → 3), Lateral-2 (3 → 4), and Posterior (4 → 1).

While most degeneration studies focus on central or posterior disc regions (Muñoz-Moya et al., 2024), the lateral circumference of the disc has been comparatively underexplored. To assess this area, we extended our analysis by slicing the disc in a mid-transverse plane (π) that bisects the nucleus pulposus (NP) and surrounding transition zone (TZ) (Figure 2A). Along the outer surfaces of the NP and TZ, we traced closed circumferential paths composed of surface nodes. For clarity, the path is partitioned into four segments (Figure 2B): Lateral-1 (1 → 2), Anterior (2 → 3), Lateral-2 (3 → 4), and Posterior (4 → 1). Then, the glucose concentration was evaluated for the tall model using GR3 properties consecutively along these segments, providing a continuous circumferential profile for both NP and TZ.

#### 2.3.3 Effect of mid-height (MH)

The influence of IVD height variations on solute concentrations, was analyzed through direct comparison of solute concentrations across different morphologies (Table 1) under specific material properties. To quantitatively assess the influence of mid-height, we computed the relative deviation of solute concentrations from the mean value across the three IVD geometries using the following expression:

$$\text{Deviation from average}\left(\%\right)=\frac{C_{i,m}-\langle C_{m}\rangle }{\langle C_{m}\rangle }\times 100,$$ (26)

where $C_{i,m}$ denotes the concentration of solute i in model m (with m= TN, MD, TL), and $\left\langle C_{m}\right\rangle$ represents the average concentration across the three models, calculated as $\left\langle C_{m}\right\rangle =\left(C_{TN}+C_{MD}+C_{TL}\right)/3$ . This analysis was conducted for each local region, PTZ, CNP, and ATZ (Figure 1), under both GR1 and GR3 material conditions.

## 3 Results

### 3.1 Metabolic transport-cell viability verification: diffusion chamber simulation

**FIGURE 3:**
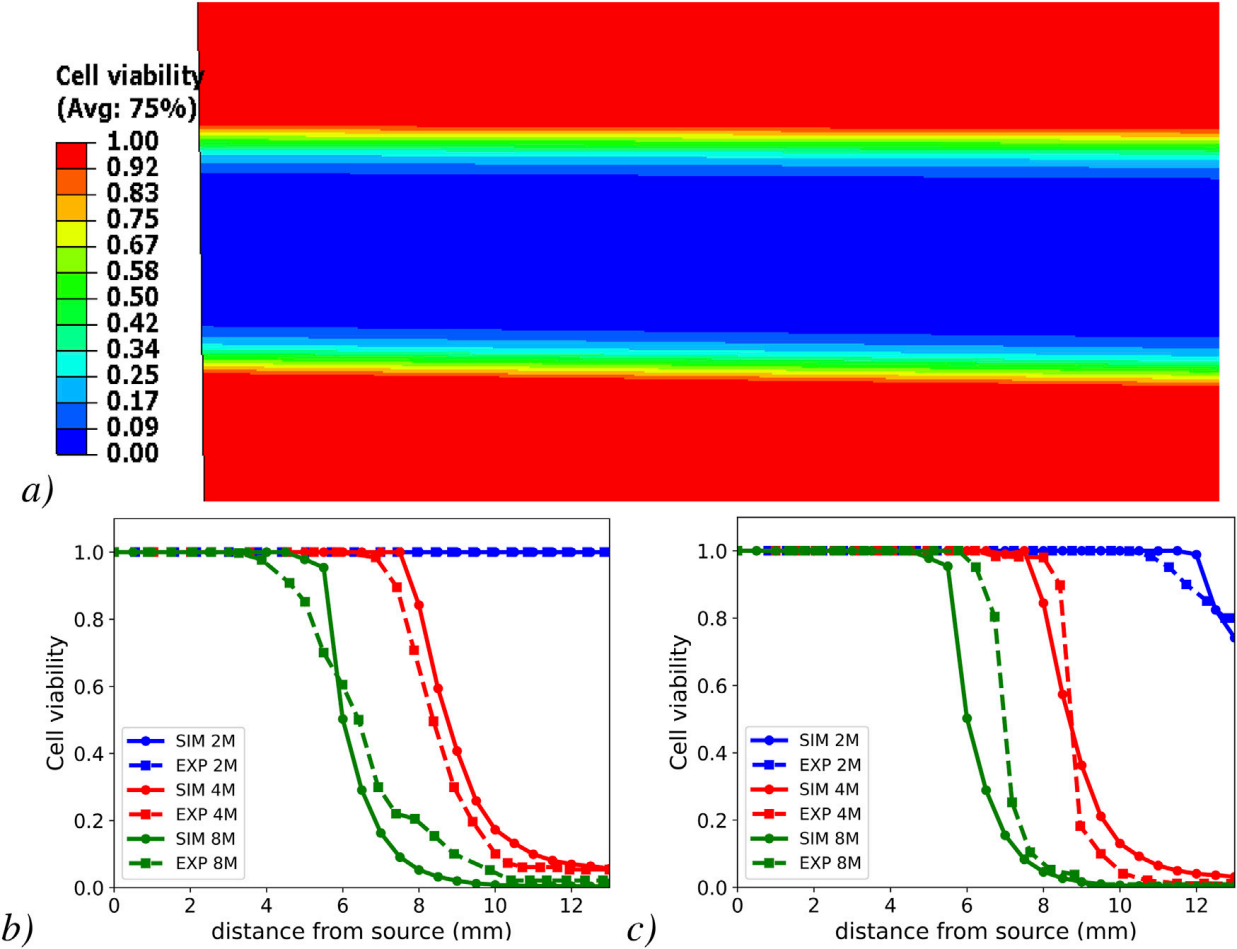
Cell viability heat map within the model diffusion chamber **(a)** and cell viability profiles in the simulated half-slice of the diffusion-chamber and their comparison with experimental results (Horner, 2001) at different cell densities. Numerical results from FEM simulation (SIM) and experiment (EXP) after day 3 **(b)** and after day 11 **(c)**.

The heatmap of cell viability (Figure 3a) within the diffusion chamber model reveals the spatial drop of cell viability, from the culture medium boundaries, to the center of the chamber. Notably, the temporal series of the spatial cell viability profiles, after 3 (Figure 3b) and 11 days (Figure 3c) of simulated diffusion, matched well the experimental results, especially at 4 million cells per $cm^{3}$ .

### 3.2 Effect of diffusivity gradient

**FIGURE 4:**
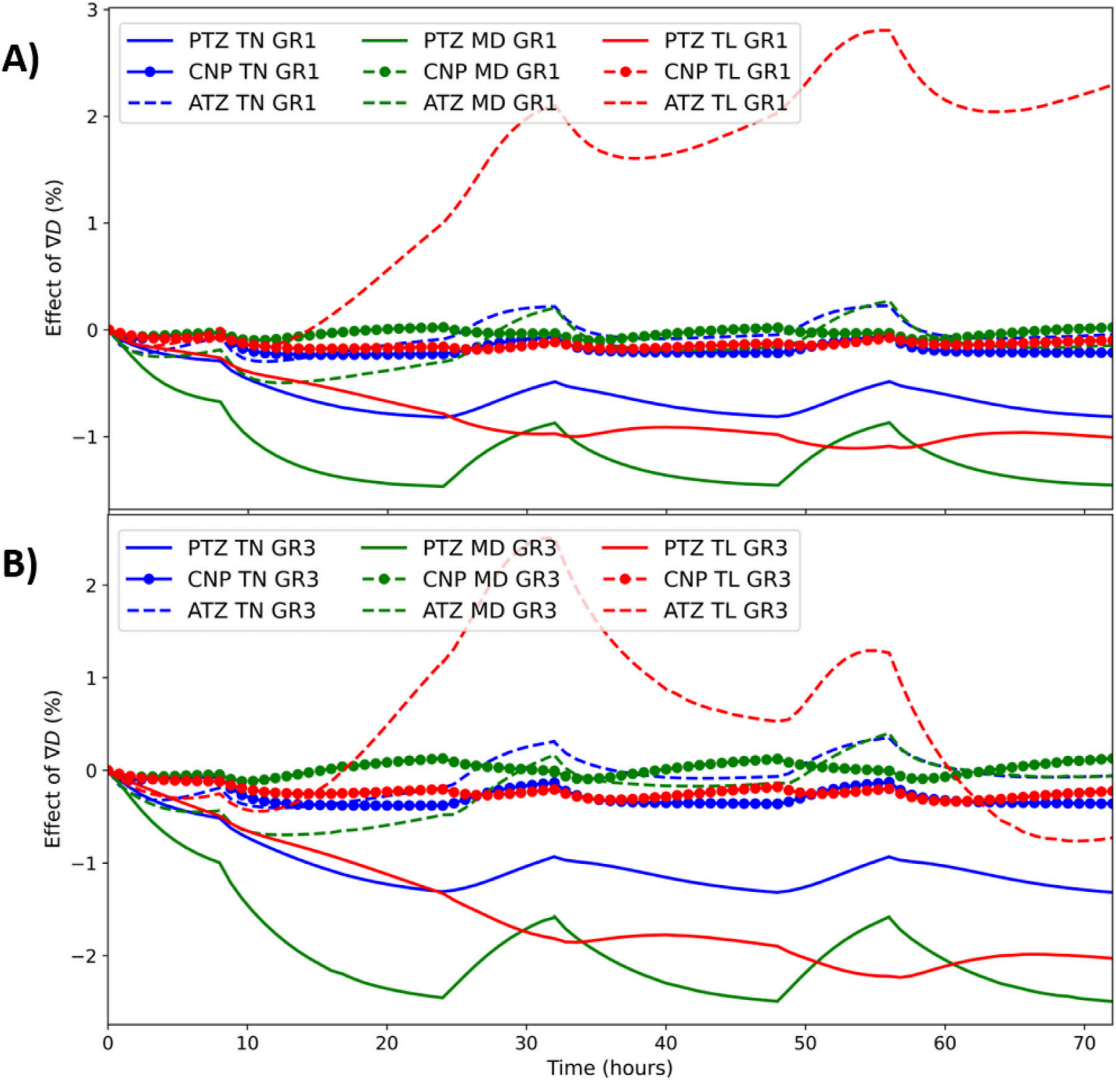
Time dependent effect of ∇D (Equation 24) on glucose concentration under GR1 **(A)**, and GR3 **(B)** at all the regions of all models.

Figure 4 displays the effect of ∇D on glucose concentrations over time, as quantified by the relative difference defined in Equation 24, for both non-degenerated (A, GR1) and degenerated (B, GR3) material properties. In both cases, the influence of ∇D remains below 3% throughout the simulation period. Similar trends were observed for oxygen and lactate, with the effect of ∇D remaining under 3%.

### 3.3 Spatio-temporal distribution of solutes under varying material properties

**FIGURE 5:**
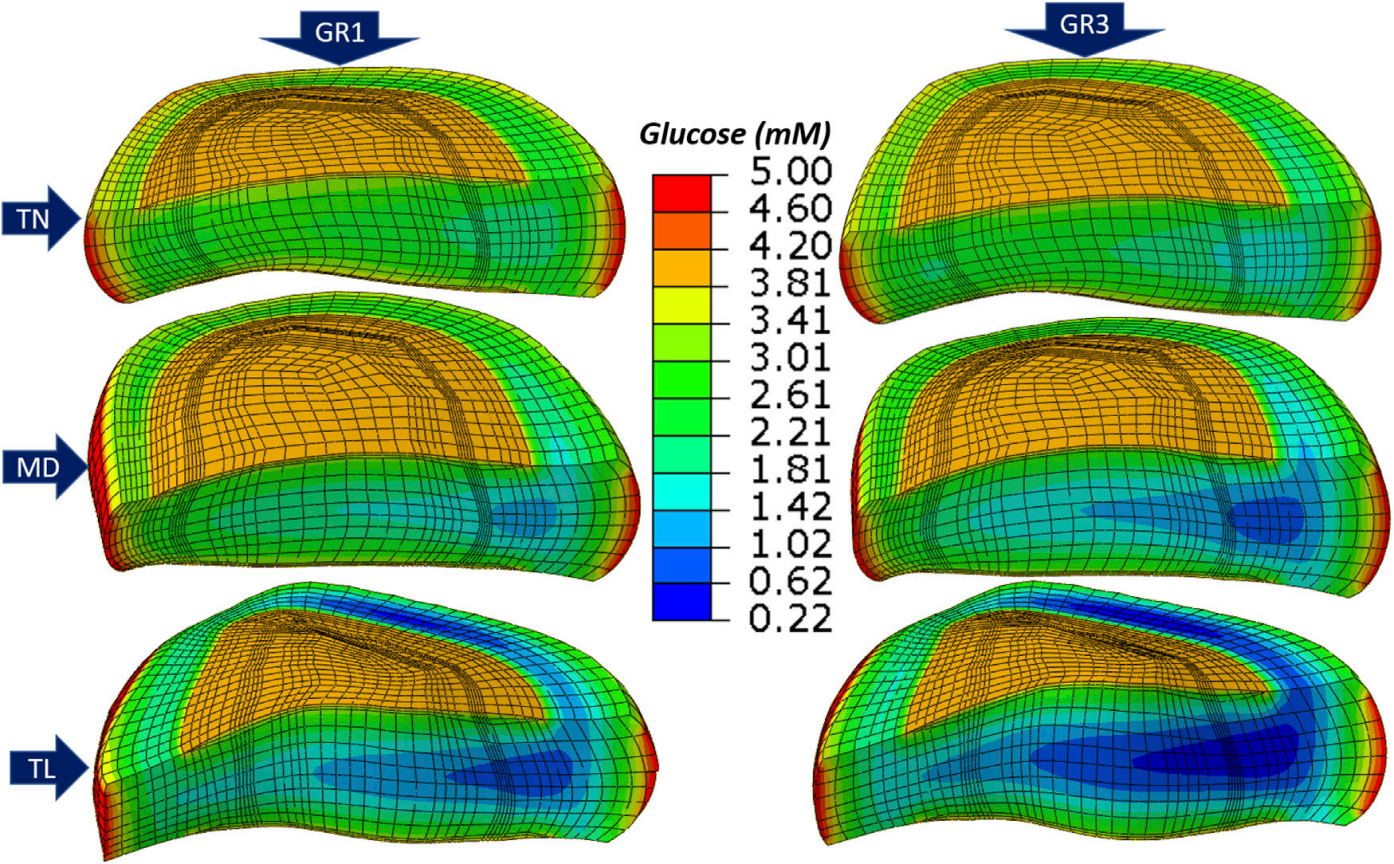
Glucose concentration for *thin* ( $1^{st}$ row), *medium* ( $2^{nd}$ row) and *tall* ( $3^{rd}$ row) models under GR1 ( $1^{st}$ column) and GR3 ( $2^{nd}$ column) tissue material properties.

Figure 5 presents glucose concentration heatmaps after 72 h of simulation across three IVD models (TN, MD, TL) and two tissue material property sets (GR1 and GR3). While simulations were performed both with and without ∇D , the results shown here correspond to ∇D=0 , since its effect on solute distribution was negligible (see Section 3.2).

Across all geometries, the TL model consistently showed the lowest glucose and oxygen concentrations and the highest lactate levels. Regionally, the anterior transition zone (ATZ) exhibited the most severe nutrient depletion and metabolite accumulation, followed by the central nucleus pulposus (CNP) and the posterior transition zone (PTZ). This spatial distribution pattern held across both material conditions. Transitioning from GR1 to GR3 led to a further decrease in oxygen and glucose concentrations and an increase in lactate levels in all regions and geometries (Figure 5). Additional results for oxygen and lactate distributions are provided in Supplementary Figures S2, S3.

**FIGURE 6:**
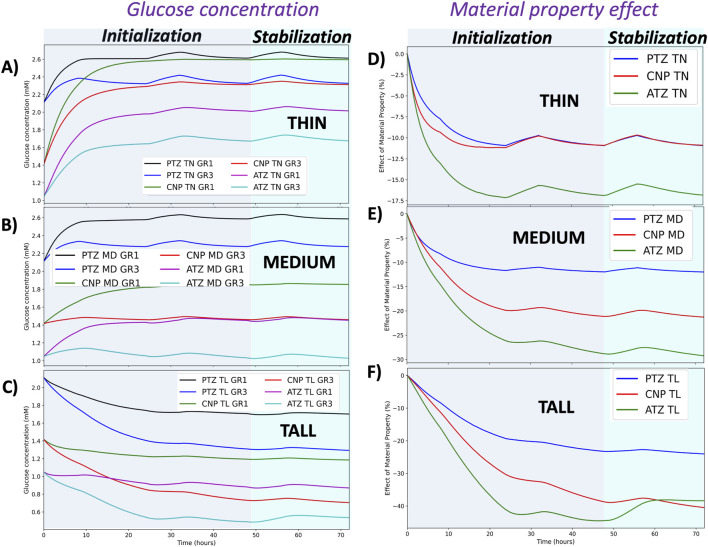
Glucose concentration profiles and impact of material properties over time. The left column **(A–C)** presents glucose concentrations in all regions and tissue material conditions for the TN **(A)**, MD **(B)**, and TL **(C)** models under GR1 and GR3 conditions. The right column **(D–F)** illustrates the relative effect of material properties (%) on glucose concentration in TN **(D)**, MD **(E)**, and TL **(F)** models across the three regions of interest (PTZ, CNP and ATZ). The background shading distinguishes simulation phases: the gray region represents the initialization phase (first two simulated days), and the light green region corresponds to the stabilization phase (final third day), during which results are considered more physiologically representative.

Figure 6 illustrates the time-dependent variations in glucose concentration for TN (A), MD (B), and TL (C) models at all regions (first column), as well as the influence of material properties (Equation 25) across the TN (D), MD (E), and TL (F) IVD models. Consistent with the spatial patterns observed in Figure 5, glucose levels progressively decline over time from the PTZ to the ATZ region, from GR1 to GR3 material conditions, and from TN to TL geometries (Figure 6, first column). Similar temporal and spatial trends were observed for oxygen concentrations, whereas lactate levels showed the opposite behavior, with greater accumulation in the ATZ region, under GR3 conditions, and in the TL model (Supplementary Figures S4, S5, first column). These temporal profiles were generated over a 72-h simulation period that incorporated a diurnal mechanical loading pattern, 8 h of rest and 16 h of activity, per day, repeated over three full cycles.

Figure 6 shows the dynamic steady-state of the three models on the third day for the glucose concentration (Figures 6A–C) and material properties (Figures 6D–F) effects, as previously shown in our prior work Malandrino et al. (2014a), Malandrino et al. (2014b). The thin and medium IVD models exhibited a marked increase in glucose and oxygen levels when compared with the initial healthy nutrient field obtained from the generic model, accompanied by a decrease in lactate concentration. In contrast, the tall disc model exhibited a sharp decline in glucose and oxygen levels in comparison with the initial healthy state, accompanied by a concurrent increase in lactate during the same period.

Despite the alternating loading pattern, temporal fluctuations in solute concentrations remained relatively small. Mild, region- and model-specific changes were observed: in the PTZ of the TN and MD models, oxygen and glucose concentrations increased slightly during rest periods, while lactate levels declined modestly. In contrast, fluctuations were minimal in the CNP and ATZ, where transport limitations and local tissue compaction are more pronounced. Across all cases, the influence of daily loading variation on solute levels remained below 5%.

The influence of material properties on glucose concentration is both model- and region-specific, with the largest relative change observed in the TL model and ATZ region, followed by the MD and TN models, and the CNP and PTZ regions, respectively (see Figure 6, second column and Table 4). Similar trends were observed for the effect of material properties on oxygen concentration, whereas lactate exhibited an inverse response. These results are detailed in Supplementary Figures S4 (second column) and S5 (second column).

**TABLE 4:** Maximum percentage change in oxygen, glucose, and lactate concentrations due to a transition from GR1 to GR3 material properties, across all local regions and IVD models.

Solute	Models								
	TN			MD			TL		
	PTZ	CNP	ATZ	PTZ	CNP	ATZ	PTZ	CNP	ATZ
Oxygen	−8%	−8%	−11%	−9%	−14%	−14%	−13%	−17%	−16%
Glucose	−10%	−10%	−17%	−10%	−17%	−27%	−21%	−40%	−45%
Lactate	12%	14%	12%	13%	14%	13%	14%	14%	11%

The impact of material property changes on solute concentrations is summarized in Table 4, based on the relative difference formula described in Equation 25. The table presents the maximum percentage change in oxygen, glucose, and lactate concentrations resulting from a shift in material properties from GR1 to GR3 across all regions (PTZ, CNP, ATZ) and IVD models (TN, MD, TL).

**FIGURE 7:**
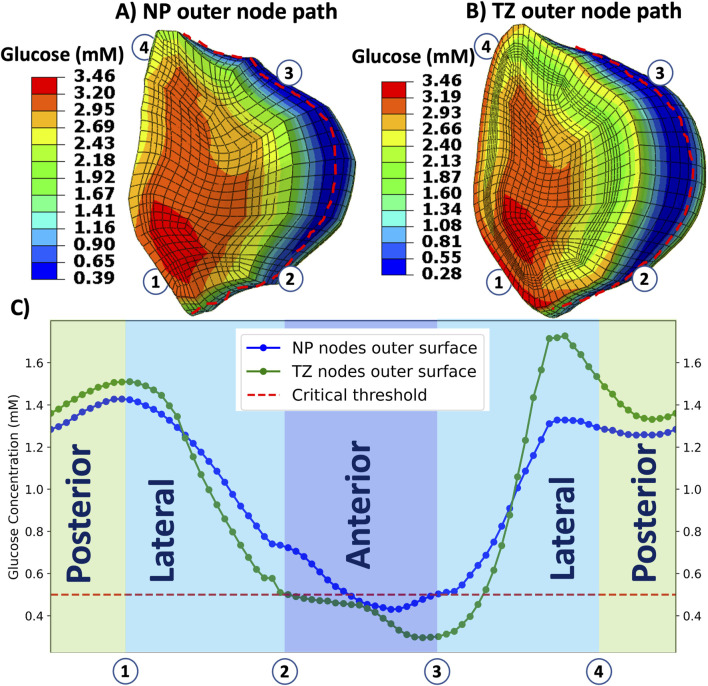
Illustrates distribution of glucose concentration over the outer surfaces of nucleus pulposus **(A)** and transition zone **(B)** and along the circumferential path **(C)** for tall (TL) IVD model under GR3 material property. Red broken curves in **(A)** and **(B)** represent the circumferential paths from where the data are collected. The horizontal dashed red line in **(C)** indicates the threshold value of glucose (0.5 mM), where below this line are nutritionally stressed regions in the nucleus pulposus and transition zone regions. Regions are divided into four segments: Lateral-1 (1 → 2), Anterior (2 → 3), Lateral-2 (3 → 4), and Posterior (4 → 1) as showed in 2B.

Figure 7 presents glucose concentrations along the circumferential mid-transverse paths (Figures 7A,B) of the outer surface of the nucleus pulposus (NP-path) and the transition zone (TZ-path) for the TL model under the GR3 condition. According to this figure (Figure 7C), the depletion of glucose in the TL model under GR3 material conditions is not localized solely to the anterior segment (2 → 3), it still extends to the anterolateral region (3 → 4).

### 3.4 Effect of mid-height (MH)

**FIGURE 8:**
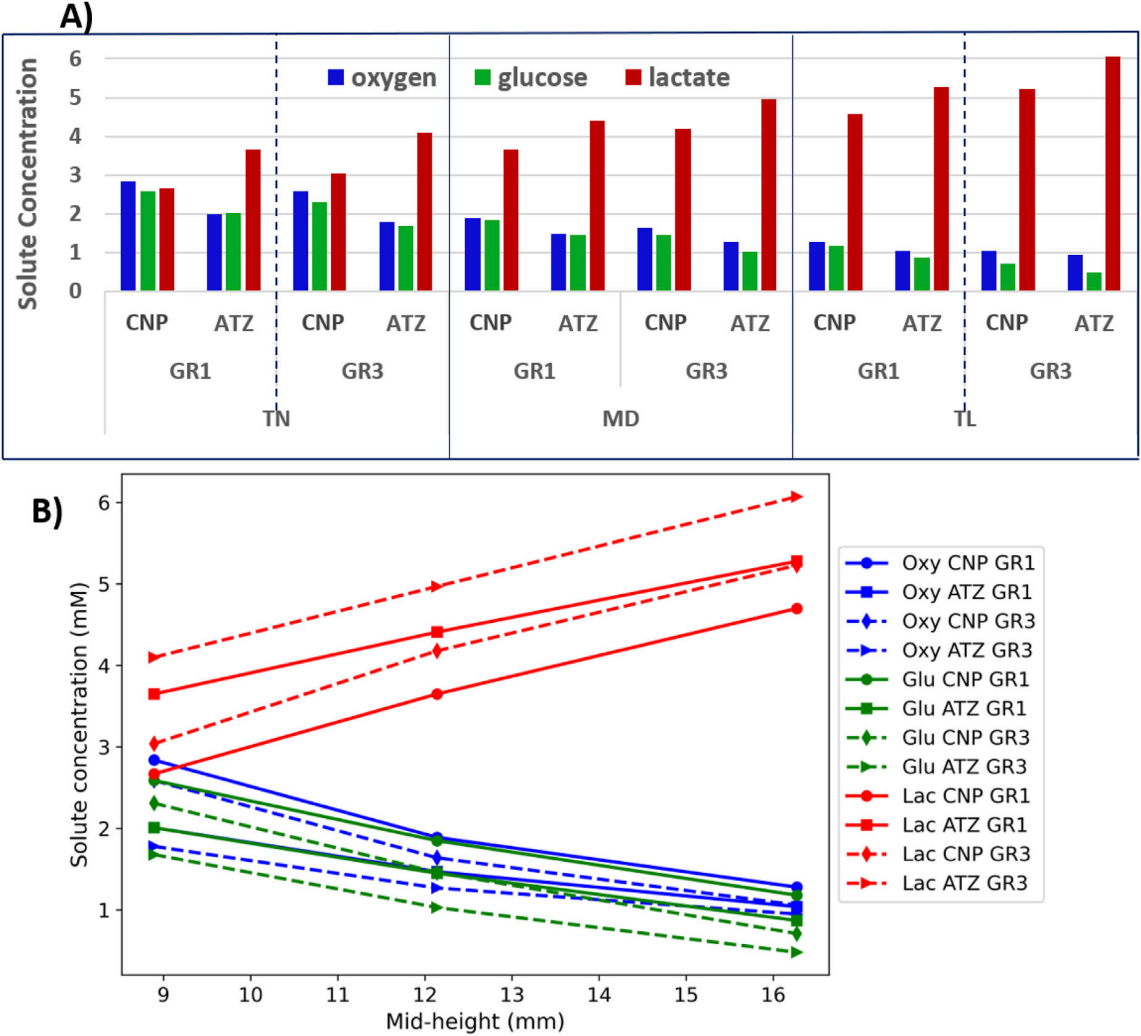
**(A)** Minimum concentrations of oxygen and glucose, and maximum concentrations of lactate; **(B)** Solute concentration as the function of disc mid-height.

Figure 8 presents an analysis of the minimum concentrations of oxygen and glucose, as well as the maximum concentration of lactate (A), and the linear dependence of these solute concentrations on disc height (B). As shown in Figure 8A, oxygen and glucose concentrations decrease progressively from left to right across each region, corresponding to a transition from TN to TL models (low to high mid-height). Conversely, lactate concentration increases along the same direction. This trend aligns with the variation in disc mid-height across the models, suggesting a linear relationship between solute concentration and disc height. This observation is further substantiated in Figure 8B, where solute concentrations are plotted directly against the mid-height of each model. Although based on only three data points, the linear trends reinforce the hypothesis that disc height is a primary determinant of solute availability within each local region.

**FIGURE 9:**
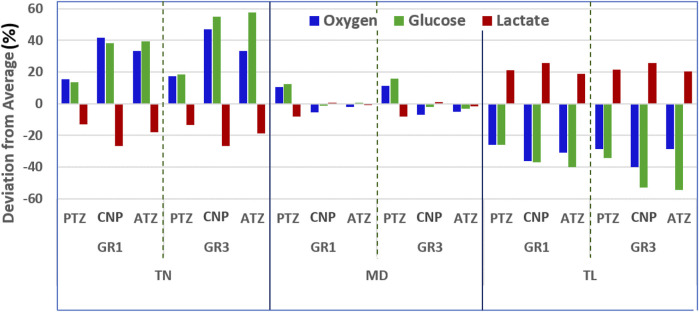
Maximum relative deviations (Equation 26) in oxygen, glucose, and lactate concentrations due to variations in disc mid-height, under GR1 and GR3 material properties after three simulated days.

Figure 9 provides a semi-quantitative illustration of how variations in disc height influence solute concentrations across regions and material conditions (Equation 26). The analysis reveals that the TN and TL models exhibit the most significant deviations from the mean, whereas the MD model shows minimal deviation, reflecting its central position in the height spectrum. The TN model shows positive deviations in oxygen and glucose concentrations, indicating elevated levels relative to the average across all geometries. In contrast, lactate exhibits negative deviations in this model. As one moves toward the TL geometry, the pattern reverses: oxygen and glucose show negative deviations, while lactate concentrations rise above the average. To complement this, Table 5 summarizes the percentage deviations (Equation 26) in oxygen, glucose, and lactate concentrations from the average across the three model geometries, specifically for TN and TL discs under both GR1 and GR3 material properties.

**TABLE 5:** Deviation from average (Equation 26) solute concentrations (%) under GR1 and GR3 conditions in TN and TL models across different disc regions.

Solute	Models											
	TN						TL					
	GR1			GR3			GR1			GR3		
	PTZ	CNP	ATZ	PTZ	CNP	ATZ	PTZ	CNP	ATZ	PTZ	CNP	ATZ
Oxygen	15.41	41.72	33.17	17.24	47.02	33.47	−25.84	−36.15	−31.03	−28.44	−40.02	−28.50
Glucose	13.56	38.18	39.36	18.02	54.95	57.77	−26.01	−36.91	−39.83	−34.23	−52.75	−54.57
Lactate	−13.06	−26.56	−17.93	−13.30	−26.66	−18.80	21.07	25.84	18.77	21.58	25.80	20.26

As presented in Table 5, a 26% reduction in disc height relative to the reference medium (MD) model led to elevated nutrient availability and diminished lactate accumulation. Under GR3 conditions, the maximum regional deviations were observed for oxygen (47.02% at CNP), glucose (57.77% at ATZ), and lactate (−26.66% at CNP). A similar, albeit less pronounced, pattern was noted under GR1 conditions, with peak deviations of 41.72% for oxygen (CNP), 39.36% for glucose (ATZ), and −26.56% for lactate (CNP) (see Table 5). Conversely, the TL model, representing a 34% increase in mid-height, exhibited marked reductions in oxygen and glucose concentrations alongside increased lactate accumulation. Under GR3 conditions, oxygen and glucose levels deviated by −40.02% (CNP) and −54.57% (ATZ), respectively, while lactate levels rose by 25.80% (CNP). This trend persisted under GR1 conditions, with deviations of −36.15% for oxygen (CNP), −39.83% for glucose (ATZ), and 25.84% for lactate (ATZ) (see Table 5).

A consistent regional pattern emerged across all simulations: the center of the nucleus pulposus (CNP) exhibited the most pronounced changes in oxygen and lactate concentrations, regardless of whether mid-height was increased or decreased. In contrast, the anterior transition zone (ATZ) consistently demonstrated the highest deviations in glucose levels.

### 3.5 Cell viability

Simulation results indicate that cell viability is influenced by a combination of factors, including disc mid-height, tissue material properties, and the local region of interest. In both TN and MD discs, as well as in TL discs with healthy material properties (GR1), cell viability remained at 100% throughout the 3-day simulation period across all examined regions.

**FIGURE 10:**
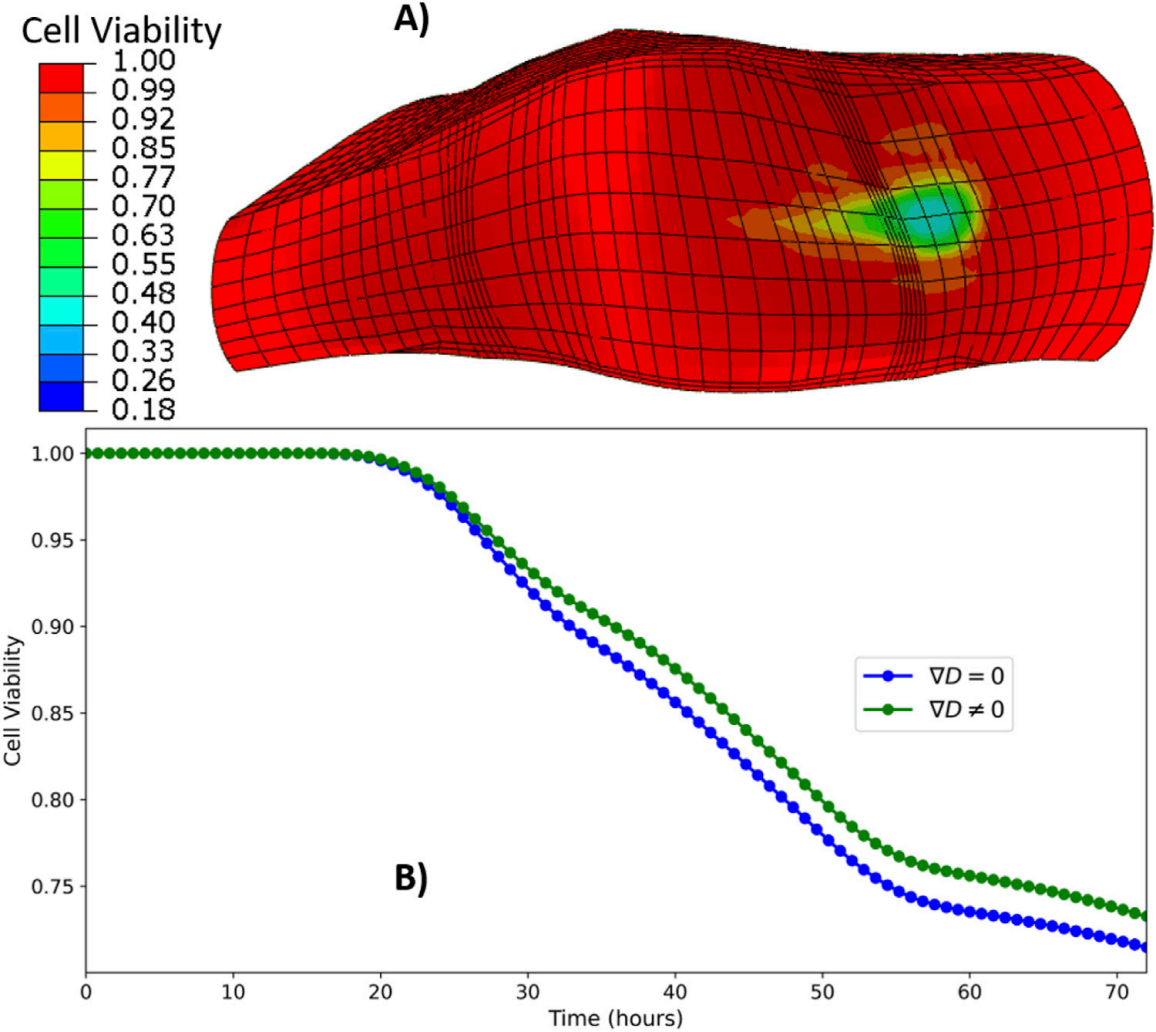
Heat map of cell viability at the end of three simulated days **(A)**, time evolution of cell viability in the ATZ region **(B)** of TL model under GR3 material property.

In contrast, under degenerated material conditions (GR3), the TL model exhibited reduced cell viability in the ATZ region. Cell death initiated as early as the first day of simulation. Figure 10A presents a heat map of cell viability after three simulated days, while Figure 10B illustrates the temporal evolution of viability in the ATZ region of the TL model under GR3 conditions. By the end of the 3-day simulation, cell survival in the ATZ region reached 72% when assuming a uniform diffusion coefficient (∇D=0) , and 73% when spatial diffusion gradients were included (∇D≠0) .

## 4 Discussion

This study utilized a poromechanics-metabolic transport-cell viability coupling model, implemented via the Finite Element Method, to investigate the intricate dynamics of nutrient transport in patient-personalized (PP) L4-L5 lumbar IVD models (Muñoz-Moya et al., 2024). By focusing on the distribution of oxygen, glucose, and lactate across varying disc geometries (TN, MD,TL) under both healthy (GR1) and degenerated (GR3) material conditions, we aimed to investigate the multifaceted interactions between structural, mechanical, and metabolic factors. The findings provided valuable information about the roles of disc morphology, physiological loading, and tissue material properties in shaping the metabolic micro-environment and local cellular viability in the IVD.

### 4.1 Model validation and biological relevance

The capacity of the reactive transport model to predict nutritional stress and corresponding cell viability, based on phenomenological sets of equations for IVD cell metabolism, was successfully validated against the independent experimental data reported by Horner (2001). Validation covered the spatio-temporal effects of diffusion-reaction transport of oxygen, glucose, and lactate, in a porous medium, for 2 cell densities that cover the cell populations in the disc tissues. The good degree of accuracy, particularly at $4\times 10^{6},{\text{cells/cm}}^{3}$ , which closely reflects the nucleus pulposus (NP) cell density *in vivo*, underlines the biological relevance of the model. This reliability to capture solute diffusion, nutrient availability, and waste removal dynamics under diverse conditions provides a robust platform to explore possible risk factors associated with spatiotemporal reactive transport of metabolites in the disc, for the times simulated hereby.

### 4.2 Spatio-temporal distribution of solutes under varying material properties

Simulation results revealed that solute distribution within the IVD is highly dependent on spatial location, time, and tissue material properties across all examined models (see Figures 5, 6; Supplementary Figures S2, S3, S4, S5; Table 4). Spatially, the ATZ of the TL model consistently exhibited the most unfavorable solute conditions, characterized by the lowest glucose and oxygen concentrations and the highest lactate accumulation. In contrast, the PTZ maintained more favorable solute profiles in all geometries, likely due to shorter diffusion distances and reduced mechanical compression.

The transient discrepancies observed in solute dynamics during the early phase of simulation, particularly the sharp increase in glucose and oxygen and drop in lactate in thinner discs (TN and MD), versus the opposite trend in the taller disc (TL), are attributed to differences in geometry relative to the reference disc used for initialization. Specifically, the initial solute concentrations for the patient-personalized models were derived from the endpoint of a 72-h transport simulation conducted on a generic disc model with a mid-height of 14.33 mm (Table 1) under identical boundary and meshing conditions. This initialization approach introduced a morphology-dependent bias at the start of the simulations: discs thinner than the generic reference exhibited shorter diffusion distances, which facilitated a rapid influx of nutrients and clearance of waste products, leading to the observed early rise in glucose and oxygen and decline in lactate (see Figure 6; Supplementary Figures S4, S5). In contrast, the taller disc, with a longer diffusion path relative to the generic model, showed an initial drop in nutrient levels and accumulation of lactate. Importantly, these transient differences that shall not be considered for the comparative analysis of the different disc morphologies diminished over time, with all models eventually reaching a dynamic steady state under diurnal loading.

Although solute concentrations evolved over the 72-h simulation period, the impact of diurnal mechanical loading remained limited. Minor increases in glucose and oxygen during rest phases and corresponding reductions in lactate were confined to the PTZ of TN and MD models (see Figure 6; Supplementary Figures S4, S5). In contrast, the central NP and anterior transition zones exhibited negligible mechanical loading dependent fluctuations. Semi-quantitatively, daily mechanical loading induced changes of less than 5% in solute levels across all conditions. These findings suggest that, under physiological conditions, structural and compositional features of the disc exert a more dominant influence on solute transport than short-term mechanical fluctuations.

This observation is consistent with several prior studies indicating that while mechanical loading can cause transient fluid movements, its overall influence on solute transport in the IVD is relatively limited compared to the dominant role of passive diffusion, particularly for small solutes such as glucose, oxygen, and lactate. Ferguson et al. (2004) demonstrated through poroelastic modeling that although fluid velocities rise during loading–unloading cycles, the resulting convective transport is minimal and insufficient to substantially enhance the delivery of small solutes within the dense extracellular matrix. Katz et al. (1986) similarly concluded from experimental analysis that diffusion remains the principal mode of solute movement due to the avascular nature and low permeability of IVD tissues, especially in the nucleus pulposus (NP). Urban et al. (1982) found that fluid flow induced by mechanical compression only marginally influences nutrient movement, and primarily near the periphery of the disc, with negligible effects in the central NP. Supporting this, Boubriak et al. (2013) reported that diurnal hydration changes can lead to transient shifts in solute availability but emphasized that the disc’s internal structure and compositional features are the dominant determinants of transport. Malandrino et al. (2014b) used a 3D finite element model to show that, under physiological diurnal loading, solute fluctuations in the central disc remained small and confined to peripheral regions, reinforcing the limited role of dynamic loading on small solute distribution. Their follow-up study (Malandrino et al., 2015b) further demonstrated that disc geometry, particularly disc height, exerts a stronger influence on nutrient gradients and predicted cell viability than loading patterns. Finally, Soukane et al. (2007) confirmed through multiphasic transport simulations that tissue parameters such as porosity and fixed charge density, rather than mechanical inputs, primarily govern the distribution of oxygen, glucose, and lactate. Collectively, these findings support our conclusion that, under physiological conditions, solute distribution in the IVD is primarily driven by structural and material properties rather than daily variations in mechanical load. Consequently, optimizing metabolic support in the IVD requires focusing on tissue morphology, health, and composition rather than relying on mechanical modulation.

Importantly, the extent to which the simulated changes in material properties impaired the metabolic transport was strongly modulated by disc geometry. In thinner disc, with less strain-induced stiffening and shorter diffusion distances, experienced relatively moderate impact on concentration changes (see Figure 6D; Supplementary Figures S4D, S5D). On the other hand, the impact on glucose concentrations in TL discs was substantial (see Figure 6F; Supplementary Figures S4F, S5F). These predictions are consistent with previous computational findings that link degeneration-induced stiffening to reduced porosity and solute transport (Malandrino et al., 2011; Galbusera et al., 2013; Malandrino et al., 2014b). The resulting environment, characterized by oxygen and glucose depletion and lactate accumulation, creates an acidic and catabolically active milieu that might impair matrix synthesis and accelerate degeneration, according to previous IVD extracellular matrix turnover model and simulations by Gu et al. (2014).

To investigate regions beyond the selected ones (PTZ, CNP, ATZ), solute distributions were mapped along circumferential paths on the NP and TZ outer surfaces as shown in Figure 7. These results revealed that glucose deficiency is not confined to anterior regions but also extends into the anterolateral regions. In the TL degenerated model, glucose concentrations fell below the viability threshold along approximately 20% of the nucleus pulposus circumference and 35% of the transition zone circumference, while posterior regions consistently maintained levels above the critical threshold (Figure 7). This broader spread of nutritional stress indicates that metabolic risk in tall degenerated discs is not strictly confined to one anatomical quadrant but may affect a wider portion of the disc regions, possibly due to asymmetric strain and diffusion barriers.

### 4.3 Effect of mid-height

Disc morphology emerged as a critical determinant of solute transport under mechanical loading. Consistent with previous studies (Magnier et al., 2009; Malandrino et al., 2015b; Malandrino et al., 2014b; Zhu et al., 2012; Jackson et al., 2011), our model revealed an inverse relationship between disc mid-height and the concentrations of oxygen and glucose, while lactate levels increased with height. These linear trends (Figure 8B) emphasize the role of diffusion distance in modulating nutrient and metabolite distribution.

Thinner discs exhibited more favorable nutrient profiles due to shorter diffusion paths and possibly higher water content, likely resulting from reduced local volumetric strain. In contrast, taller discs experienced lower oxygen and glucose concentrations and greater lactate buildup, conditions unfavorable for cellular homeostasis. This was likely attributed to increased diffusion distance, strain-induced tissue compaction, and radial NP expansion, which collectively hinder solute transport.

These geometric effects are further mediated by the mechanical behavior of the annulus fibrosus (AF), which resists radial expansion of the NP. As a result, localized compaction near the NP–AF boundary, especially in taller discs, reduces water content and impairs solute exchange in the adjacent transition zones (O’Connell et al., 2012; Iatridis et al., 1998; Sher et al., 2017). Such strain-driven porosity changes amplify spatial heterogeneity in nutrient distributions, a phenomenon consistently observed in previous studies (Jackson et al., 2011; Malandrino et al., 2014b; Zhu et al., 2012).

Our results align with those of Malandrino et al. (2015b), who demonstrated that disc geometry can critically affect nutrient availability (see Figures 8A, 9). In particular, tall discs with mid-heights comparable to our TL configuration exhibited nutrient levels falling below viability thresholds. This effect was most pronounced in the anterior transition zone (ATZ), where limited permeability and higher metabolic demands converge (Sher et al., 2017).

Similarly, Motaghinasab et al. (2014) highlighted the size dependence of solute penetration in systemically delivered drugs, reporting that larger discs exhibit extended diffusion times and reduced permeability due to tissue consolidation. Additional study (Schmidt et al., 2016) confirms that increased disc height also influences mechanical strain patterns, which in turn modulate matrix porosity and interstitial fluid flow. These interactions restrict nutrient supply while promoting metabolic waste accumulation, increasing the risk of cell death and disc degeneration.

The medium-sized disc (MD) represented a physiologically favorable balance, exhibiting solute concentrations close to the overall average (Figure 9). Simulations showed that reducing disc mid-height by approximately one-third relative to MD increased oxygen and glucose concentrations by over 30% and decreased lactate levels by at least 20% in both the CNP and ATZ. Conversely, increasing mid-height by the same proportion resulted in over 30% reductions in oxygen and glucose, along with comparable increases in lactate accumulation. These effects were observed under both GR1 and GR3 material properties, with stiffer (GR3) tissue exacerbating transport limitations. Together, these results demonstrate that deviations from intermediate disc geometry significantly alter the metabolic microenvironment and may elevate the risk of degeneration, especially through GR3-level tissue changes.

Beyond global disc height, regional morphology, particularly antero-posterior asymmetry, also influenced nutrient transport (see Supplementary Figure S6). Discs with identical mid-heights but differing anterior and posterior heights showed clear differences in solute distribution, especially in the ATZ. These differences stem from localized deformation patterns that affect porosity and thus transport, consistent with findings in Muñoz-Moya et al. (2024). Taller regions, often anterior, are subject to greater axial strains under follower loads (Schmidt et al., 2007), due to geometric nonlinearity and strain-dependent stiffening. This leads to local tissue consolidation, increased NP expansion, and reduced permeability in the adjacent transition zone.

These results show how regional disc morphology intricately shapes transport dynamics within the IVD. They suggest that local structural variations should be accounted for when assessing disc health. Studies have also shown a positive correlation between body height and disc height, with taller individuals generally having taller intervertebral discs (Salamon et al., 2017). The present study suggests that taller individuals may face a higher risk of metabolic imbalances. This could potentially contribute to disc degeneration.

### 4.4 Cell viability

The findings demonstrated the relationships among disc geometry, material properties, and nutrient transport in maintaining cell viability within intervertebral discs. For thin and medium-sized discs, with both GR1 and GR3 material properties, as well as the tall IVD with GR1 material properties, nutrient diffusion was sufficient to sustain 100% cell viability. This is observed across all considered regions of the IVD over 3-day cycles of mechanical loading. This outcome differs from the results of Malandrino et al. (2015b), who also reported cell death in the center of the NP of their tallest (15.2 mm) IVD model, even with healthy material properties. Arguably, the authors did not have a composition-based formulation of the disc tissue beyond the AF fibres, and they did not consider strain-dependent osmotic pressurization but a constant osmotic pressure, which might not be as helpful to retain the extrafibrillar water under mechanical loads. Such interpretation is consistent with the findings by Muñoz-Moya et al. (2024), where mid disc height stood for a top morphological feature that affects the control of extrafibrillar water in the center of the NP, with a tissue constitutive model similar to the one used here. Overall, the present results emphasize the importance of maintaining material composition to support adequate nutrient supply and waste removal through diffusive transport.

However, the challenges posed by degeneration became evident in tall IVDs with mildly compromised material properties (GR3). In these cases, the ATZ regions were particularly susceptible to nutrient deficits, with significant cell death observed as early as in the first day of simulation (Figure 10). This is in line with the reports related to the effect of disc size and material property on cell survival (Malandrino et al., 2015b; Gu et al., 2014). As stated in the result section, including ∇D in the diffusion-reaction framework (Equation 10) did not lead to any considerable change in cell viability compared to that without ∇D (Figure 10).

While the study offers a structured understanding of how these factors affect nutrient availability and cellular viability, some limitations are acknowledged. The model’s assumptions of uniform tissue properties, such as initial water content, cell density, and fixed charge density, as well as the exclusion of factors like inflammatory cytokines and structural protein-degrading enzymes, which are key features of degenerated IVD, may reduce its ability to capture localized variations or simulate long-term degenerative effects. Additionally, the simulations were limited to IVD models with minimal wedging, where the mid-height adequately represents the overall geometry. Incorporating greater local variability, as recently done in mechanical simulations by Muñoz-Moya et al. (2024), would enrich the interpretation of spatial transport dynamics. However, such integration remains computationally expensive under the current framework, where a 3-day simulation of an IVD model requires over 288 h on high-performance computing infrastructure. Future studies, by coupling the current finite element models with existing systems biology models of IVD cell activity (Baumgartner et al., 2021; Tseranidou et al., 2025), should address these gaps, allowing for dynamic descriptions of biochemical factors and their spatial heterogeneity.

## 5 Conclusion

This study presented a comprehensive evaluation of how IVD morphology, tissue composition, and mechanical deformation interact to regulate nutrient transport and cellular viability. By leveraging poro-mechanical finite element models of three PP L4-L5 IVD geometries, we systematically quantified the influence of disc mid-height, degeneration-dependent tissue properties, and strain-induced diffusivity gradients (∇D) on solute distribution under physiological loading.

The results demonstrated that disc morphology and tissue material properties are the principal regulators of solute availability. Thinner and medium-sized discs consistently exhibited favorable metabolic profiles, with oxygen and glucose levels maintained above critical thresholds and reduced lactate accumulation. In contrast, taller discs, particularly those with degenerated material properties (GR3), showed marked declines in oxygen and glucose concentrations, exceeding 30% , and lactate increases of ≥20% , especially in the anterior transition zone. These changes reflected the compounded effects of increased diffusion distances, strain-induced reductions in porosity, and compromised matrix permeability. Consequently, glucose levels in tall-degenerated discs fell below the viability threshold, leading to cell death in vulnerable regions.

The role of degeneration was further underscored by the strong modulation of solute profiles by tissue material properties. Stiffer, less hydrated tissues (GR3) exhibited up to 45% reductions in glucose. These reaffirmed the importance of compositional integrity in preserving nutrient diffusion and highlight the synergistic threat posed by unfavorable geometry and degeneration.

Although physiological loading cycles induced minor temporal variations in solute concentrations, slightly improving nutrient levels during rest and reducing them during activity, their relative effect remained below 5% across all regions and models. Likewise, inclusion of the ∇D term in the reaction-diffusion equation had negligible impact on overall concentration profiles and cell viability outcomes. These calculations suggest that while mechanical loading and strain-dependent diffusivity may fine-tune solute gradients, they are secondary to the dominant influences of morphology and material property. Accordingly, the present study shall not question the value of previous models and simulations that used linear mechano-transport coupling approximations.

Taken together, these findings highlight the biomechanical and structural parameters that most critically shape nutrient availability and cellular health in a spatially and solute-specific manner. In particular, disc mid-height and tissue degeneration emerged as key drivers of metabolic imbalance with potential risk factors for region-specific disc degeneration. From a translational standpoint, this work supports the development of personalized IVD models incorporating PP geometry and material characteristics as a basis for improved diagnostic biomarkers of IVD morphology that can contribute to the assessment of the risk of degeneration or incremental degeneration of lumbar IVDs.

## Data Availability

The original contributions presented in the study are included in the article/Supplementary Material, further inquiries can be directed to the corresponding author.

## Footnotes

1 The repository of 169 PP FE models of the IVD created for the scientific community (Muñoz-Moya et al., 2024) is available in (Muñoz-Moya et al., 2023), accessible through our online user interface SpineView: https://ivd.spineview.upf.edu/.
