# Supplementary material for: Influence of disc height and strain-dependent solute diffusivity on metabolic transport in patient-personalized intervertebral disc models
Source: Front Bioeng Biotechnol. 2025 Sep 5;13:1651786. doi: 10.3389/fbioe.2025.1651786 (PMC12447076; doi:10.3389/fbioe.2025.1651786)
Supplement: Supplementary file 1 [file DataSheet1.pdf]

Influence of Disc Height and Strain-Dependent Solute Diffusivity on Metabolic Transport in Patient-Specific Intervertebral Disc Models.

Zerihun G. Workineh [1,*], Estefano Muñoz-Moya [1], Carlos Ruiz Wills [1], Dimitrios Lialios [2] and Jérôme Noailly [1]

[1] *Biomechanics and Mechanobiology, Department of Engineering, Universitat Pompeu Fabra, Barcelona, Spain*
[2] *Barcelona Supercomputing Center, Barcelona, Spain*

Correspondence*:
Zerihun G. Workineh
zerihungetahun.workineh@upf.edu

SUPPLEMENTARY INFORMATION

1 S_1: NUMERICAL IMPLEMENTATION

The numerical framework developed by Malandrino et al. has been applied in this study as stated in the main manuscript, integrating a poromechanical finite element (FE) model with transport FE models to account for the interconnected metabolic processes within the IVD. In this approach, the solute transport models rely on the deformation history of the poromechanical model to continuously update concentration gradients over time. The transport models are solved sequentially at each time step, incorporating a cell viability model to regulate cell density and subsequently influence solute consumption and production. A schematic representation of this methodology is shown in Figure S1 for further clarification.

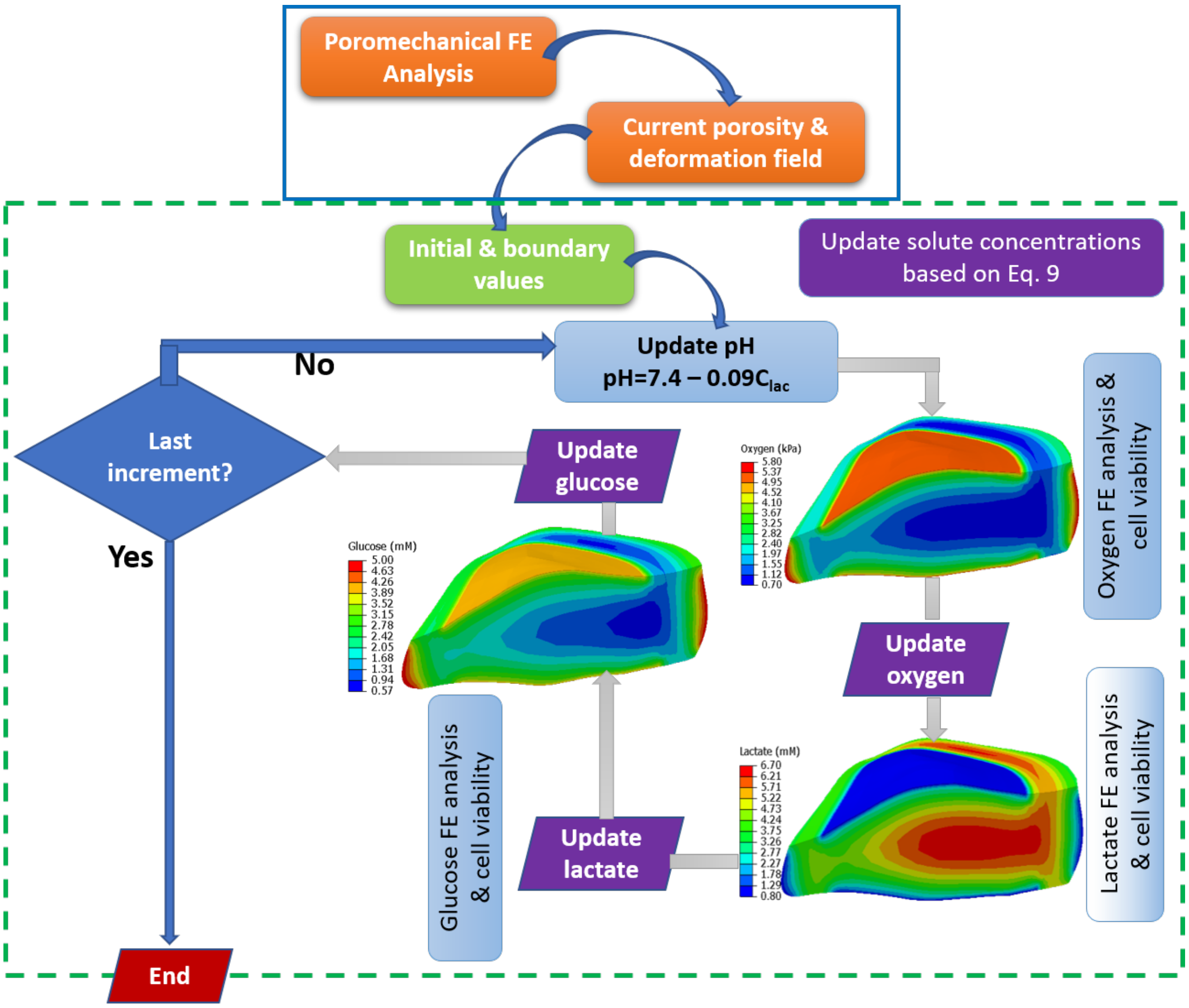

Figure S1. Schematic representation of the coupled poromechanical-transport-cell viability finite element (FE) models, integrating solute transport and metabolic reactions within the intervertebral disc (IVD). The poromechanical FE model (solid blue box) computes the deformation-dependent fields of porosity and strain, which dynamically influence the transport FE models (dashed green box). At each time step, solute transport is updated sequentially (indicated by arrows) based on Equation 9 in the main manuscript, adjusting glucose, oxygen, lactate, and pH levels. The metabolic feedback loop accounts for cell viability, modifying solute consumption and production rates. Heatmaps visually represent solute distribution within the IVD, enhancing the schematic's interpretability.

2 S_2: RESULTS

Spatio-tmeporal dependent metabolic concentrations

Figure S2 and S3 show heatmap of oxygen and lactate,respectively, concentrations across the different regions of model IVDs.

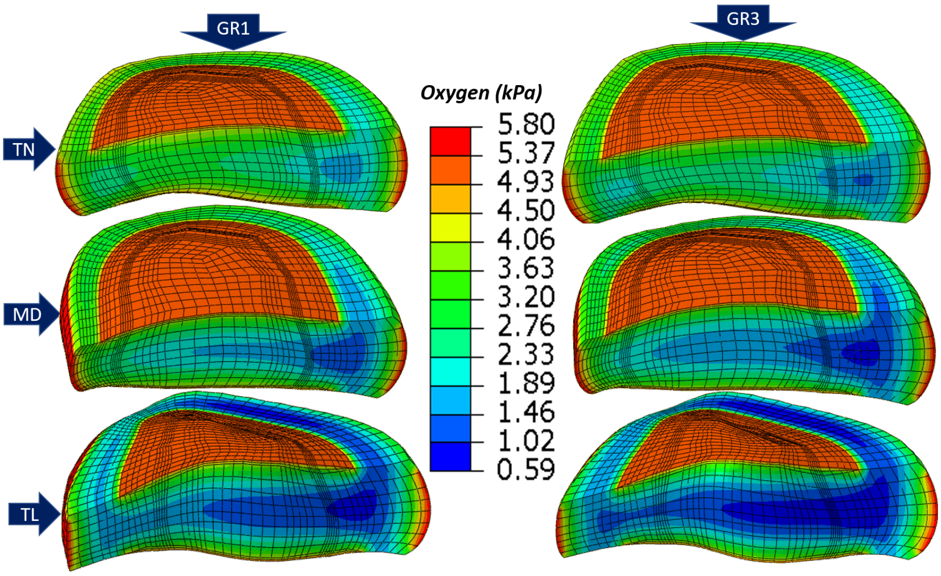

Figure S2. Heatmap of oxygen concentration for *thin*, *medium* and *tall* IVD models at GR1 and GR3 material properties at the end of 3 simulated days.

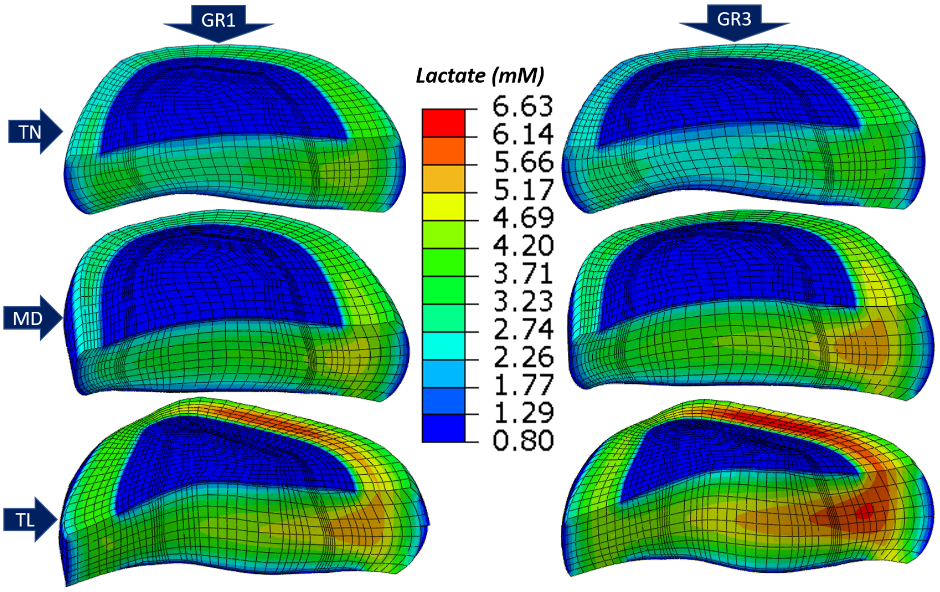

Figure S3. Heatmap of lactate concentration for *thin*, *medium* and *tall* IVD models at GR1 and GR3 material properties at the end of 3 simulated days.

The time dependent concentrations of oxygen and lactate together with the time dependent material property effect for all models at all regions are shown in Figure S4 and Figure S5, respectively. These Figure clearly show strong dependence of solute concentrations on morphology, material property and local regions.

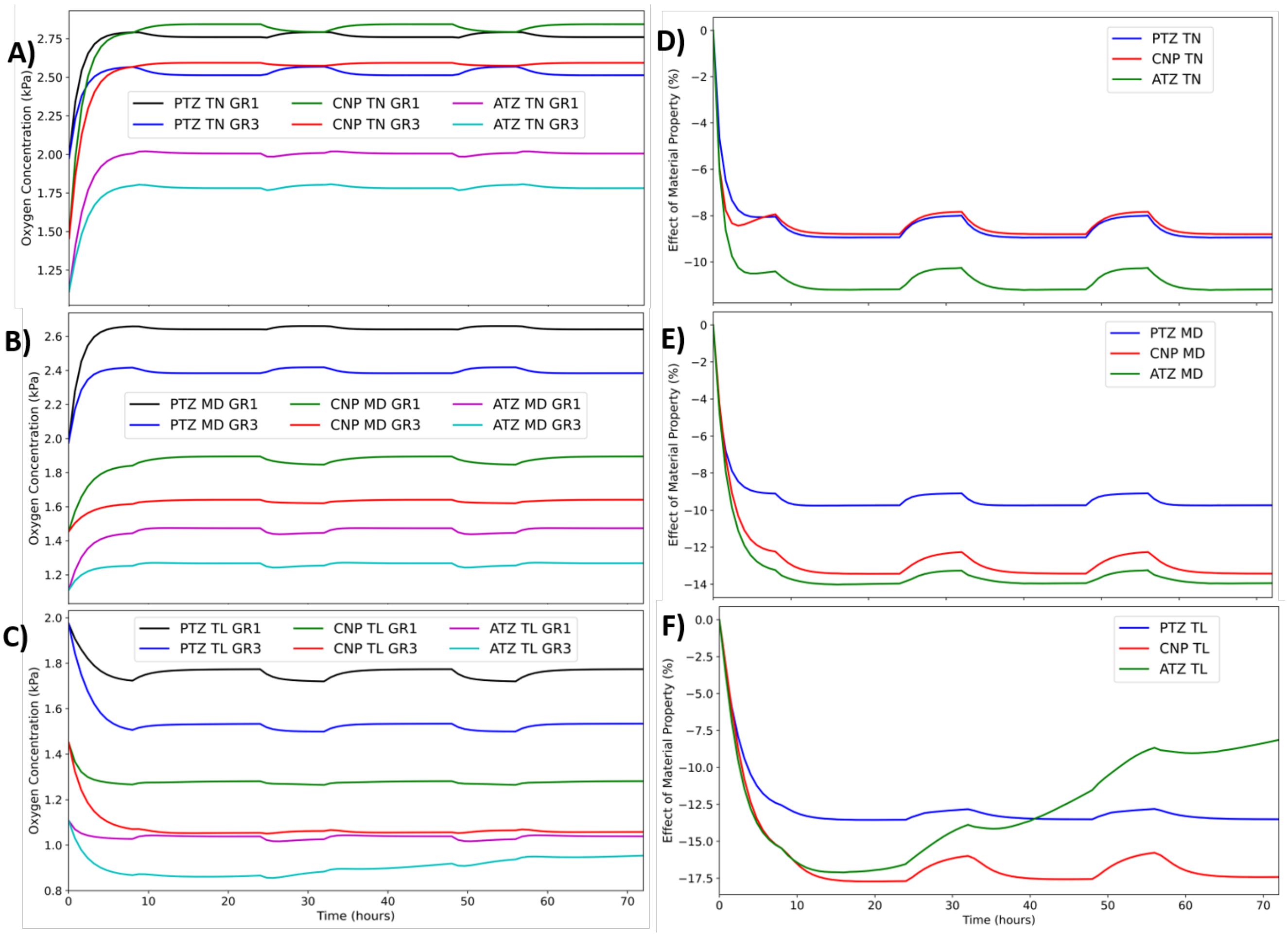

Figure S4. Oxygen concentration profiles and the impact of material properties as the functions of time. The first column (A–C) shows oxygen concentrations measured at all regions and tissue material conditions for TN (A), MD (B), and TL (C) regions under. The second column (D–F) illustrates the effect of material properties on oxygen concentrations in the TN (D), MD (E), and TL (F) models across the three regions of interest.

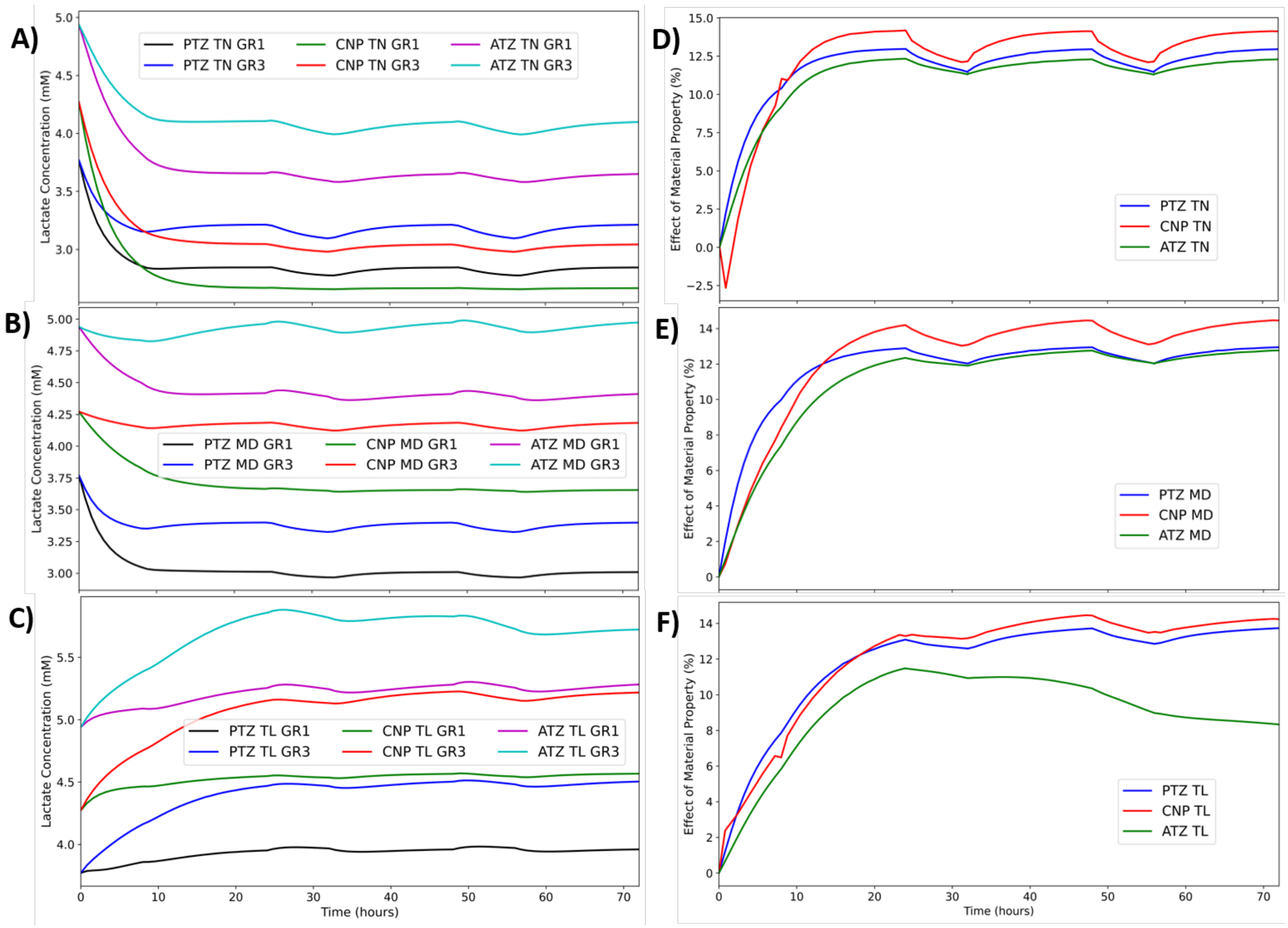

Figure S5. Lactate concentration profiles and the impact of material properties as the functions of time. The first column (A–C) shows lactate concentrations measured at all regions and tissue material conditions for TN (A), MD (B), and TL (C) regions under. The second column (D–F) illustrates the effect of material properties on lactate concentrations in the TN (D), MD (E), and TL (F) models across the three regions of interest.

16 **Glucose Concentration in wedge-shaped model**

The heatmap of glucose concentrations for the wedge-shaped patient-specific model is presented in Figure S6. Key geometric features of this model include a mid-height of 8.04 mm, an anterior height of 23.09 mm, and a posterior height of 9.89 mm. As shown in the Figure, glucose concentrations in the anterior (ATZ) region are significantly lower compared to those in the same region of a different model with the same mid-height (see Figure 5 in the main text). This reduction is attributed to the increased diffusion distance. This change not only impacts the glucose levels in the ATZ region but also affects concentrations in other regions, such as the CNP.

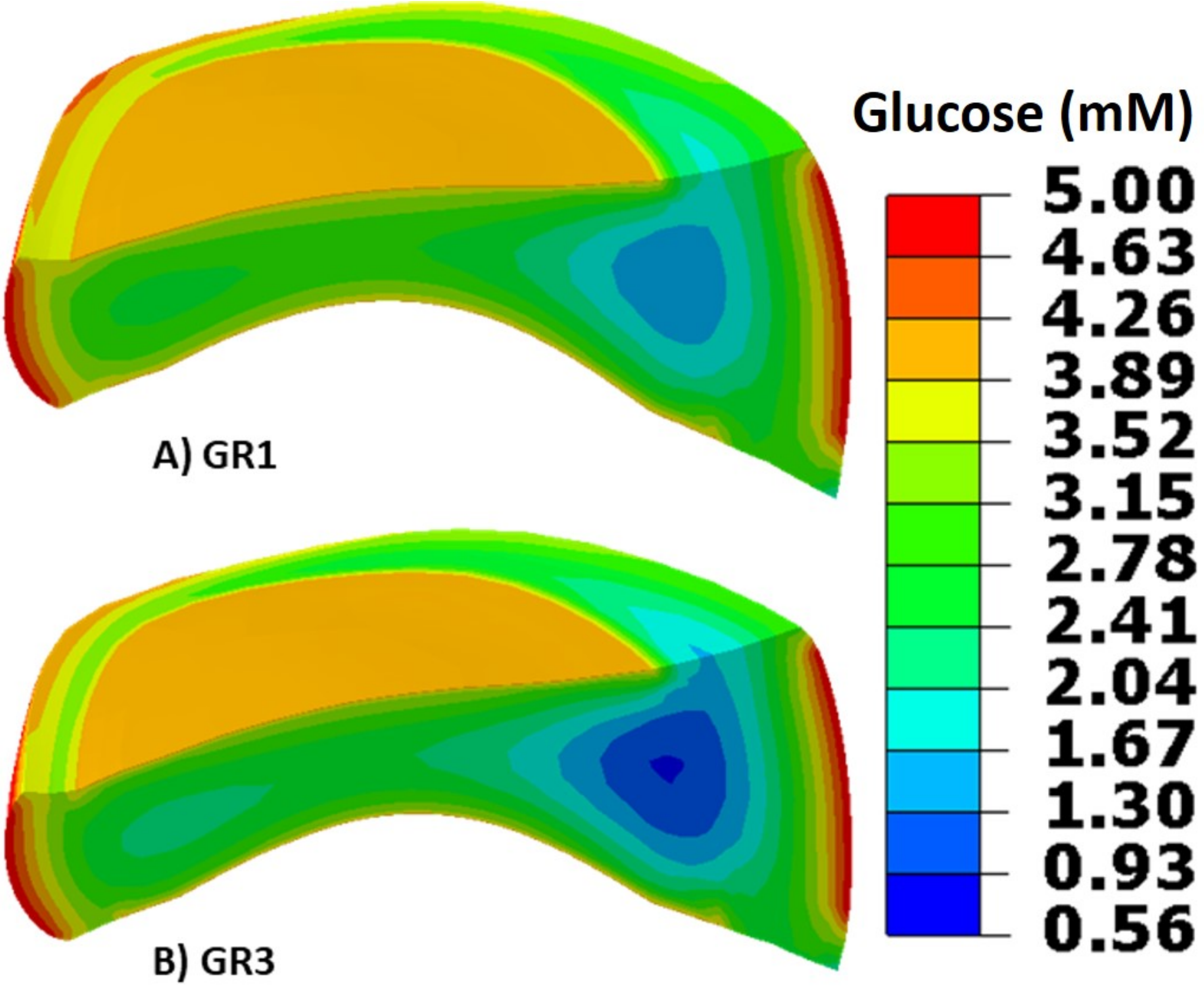

Figure S6. The heatmap of glucose concentrations in the wedge-shaped IVD model under GR1 (A) and GR3 (B) conditions.
